# Wedelolactone, a Novel TLR2 Agonist, Promotes Neutrophil Differentiation and Ameliorates Neutropenia: A Multi‐Omics Approach to Unravel the Mechanism

**DOI:** 10.1002/advs.202509807

**Published:** 2025-12-23

**Authors:** Long Wang, Zhichao Li, Tianci Hu, Qinyao Li, Linwei Zhang, Xinyue Mei, Xiao Qi, Sheng Liu, Weijie Kong, Jiesi Luo, Anguo Wu, Feihong Huang, Sirui Li, Shuang Dai, Chunxiang Zhang, Rong Li, Jianming Wu

**Affiliations:** ^1^ Department of Pharmacology School of Pharmacy Southwest Medical University Luzhou Sichuan 646000 China; ^2^ State Key Laboratory of Southwestern Chinese Medicine Resources School of Pharmacy Chengdu University of Traditional Chinese Medicine Chengdu Sichuan 611137 China; ^3^ School of Basic Medical Sciences Southwest Medical University Luzhou Sichuan 646000 China; ^4^ Key Laboratory of Medical Electrophysiology Sichuan Key Medical Laboratory of New Drug Discovery and Druggability Evaluation Luzhou Key Laboratory of Activity Screening and Druggability Evaluation for Chinese Materia Medica Southwest Medical University Luzhou Sichuan 646000 China; ^5^ Laboratory for Cardiovascular Pharmacology of Department of Pharmacology The School of Pharmacy Southwest Medical University Luzhou Sichuan 646000 China; ^6^ Drug Discovery Research Center Southwest Medical University Luzhou Sichuan 646000 China

**Keywords:** neutropenia, nutrophil differentiation, TLR2, wedelolactone

## Abstract

Neutropenia, a common complication in cancer patients undergoing radiotherapy, heightens the risk of infection and mortality, with limited treatment options. This study investigates wedelolactone (WED), a natural coumarin, as a potential therapeutic agent. WED is found to promote neutrophil differentiation and enhance bactericidal function in vitro. Its in vivo efficacy is validated in radiation‐induced neutropenic mouse and zebrafish models, where it facilitates rapid recovery of leukocyte and neutrophil levels. An integrated approach using the GEO database, RNA sequencing, molecular docking, and Drug Affinity Responsive Target Stability (DARTS) assays identifies TLR2 and its downstream MAPK signaling pathway as essential for WED's anti‐neutropenic effects. DARTS confirms significant binding of WED to TLR2. Knockdown of TLR2 with siRNA or inhibition of TLR2 with C29 reduces WED‐induced neutrophil differentiation, MEK1/2 and ERK1/2 phosphorylation, and expression of transcription factors (PU.1, CEBPβ). Similarly, ERK1/2 inhibition by SCH772984 impairs WED‐induced neutrophil differentiation and bactericidal activity, decreasing PU.1 and CEBPβ expression without affecting TLR2 levels. These findings position TLR2 as a key therapeutic target for neutropenia, with WED effectively promoting neutrophil differentiation via TLR2 and MEK/ERK pathway activation. This study highlights the therapeutic potential of targeting TLR2 to alleviate neutropenia and underscores the utility of a multi‐omics approach in uncovering drug mechanisms.

## Graphical abstract

Wedelolactone (WED), a natural TLR2 agonist, promotes neutrophil differentiation and enhances bactericidal function, offering a potential therapeutic strategy for neutropenia. Using a multi‐omics approach, this study reveals that WED activates the TLR2/MEK/ERK pathway, upregulating key transcription factors (PU.1, CEBPβ) to drive neutrophil development. These findings highlight WED's potential as a novel treatment for neutropenia.

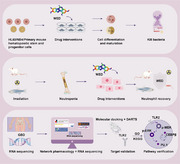

## 1 Introduction

Chemotherapy and radiotherapy, while effective in cancer treatment, often induce neutropenia, a debilitating condition characterised by a severe reduction (< 1.5 × 10^9^/L) in circulating neutrophils. This complication increases susceptibility to infections and often necessitates treatment interruption.^[^ ^1^, ^2^ ^]^ Neutrophils, the most abundant white blood cells (WBCs) (comprising up to 70% of circulating leukocytes), serve as the body's first line of defence against invading pathogens.^[^ ^3^ ^]^ They employ several mechanisms to combat infections, including phagocytosis, reactive oxygen species (ROS) production, antimicrobial protein secretion, and neutrophil extracellular traps.^[^ ^4^, ^5^ ^]^ Neutrophil development is a tightly orchestrated process involving a sequential series of differentiation stages: myeloblasts, promyelocytes, myelocytes, metamyelocytes, and mature segmented neutrophils. This differentiation is regulated by a constellation of transcription factors.^[^ ^6^ ^]^ Key players in this regulatory network, including PU.1, C/EBP‐α, C/EBP‐β, and c‐Myb, exert profound control over the differentiation program that shepherds bone marrow precursor cells toward the neutrophil lineage.^[^ ^7^, ^8^ ^]^

Toll‐like receptors (TLRs) are an important family of type I transmembrane pattern recognition receptors within the innate immune system. These receptors recognize pathogen‐associated molecular patterns (PAMPs) expressed by microbes, and trigger downstream signaling cascades that activate immune cells and stimulate cytokine production.^[^ ^9^, ^10^ ^]^ Notably, TLR2 activation in neutrophils leads to the shedding of L‐selectin (CD62L) and upregulation of CD11b expression.^[^ ^11^ ^]^ Recent studies in crab‐eating macaques suggest that TLR2 agonists may serve as potential therapeutic agents to mitigate chemotherapy‐induced neutropenia.^[^ ^12^ ^]^ These findings highlight the potential of small‐molecule TLR2 agonists as an innovative approach for treating neutropenia.

Currently, granulocyte colony‐stimulating factor (G‐CSF) is the primary treatment for neutropenia induced by radiotherapy and chemotherapy, as it promotes neutrophil production.^[^ ^13^, ^14^ ^]^ However, its clinical utility is tempered by the potential for rare but severe adverse effects, including spleen rupture, allergic reactions, and vascular events. Moreover, accumulating evidence suggests that G‐CSF may compromise neutrophil function.^[^ ^15^ ^]^ Early studies have reported impaired chemotaxis and bactericidal activity against *Staphylococcus aureus* (*S. aureus*).^[^ ^16^ ^]^ Additionally, neutrophils derived from G‐CSF‐induced CD34^+^ cells may exhibit deficiencies in bacterial killing, potentially due to a lack of fully matured granules.^[^ ^17^ ^]^ These findings underscore the pressing need to develop new therapeutic strategies with improved efficacy and reduced toxicity for managing neutropenia.

The rising prominence of natural products research has spurred the exploration of bioactive components, owing to their abundance and accessibility.^[^ ^18^ ^]^ Their intricate structural diversity and pleiotropic biological activities make them valuable resources for the discovery of novel therapeutic agents.^[^ ^19^ ^]^ Wedelolactone (WED), a natural coumarin extracted from *Ecliptae Herba* (Mohanlian) and *Wedelia calendulacea*, exhibits an array of beneficial properties, including anti‐inflammatory, anti‐fibrotic, and anti‐diabetic effects.^[^ ^20^, ^21^ ^]^ In a previous study, we developed an innovative computational method to identify novel therapeutic agents with haematopoietic activity. This approach, which combines three deep learning algorithms—recurrent neural networks (RNNs), deep neural networks (DNNs), and hybrid neural networks (RNNs+DNNs), allowed us to screen compounds from the FDA‐approved drug library.^[^ ^22^ ^]^ Through this process, we identified WED as a promising candidate for treating thrombocytopenia.^[^ ^22^ ^]^ Notably, WED's structural divergence from conventional drugs, along with its ability to promote platelet production, made it an attractive candidate for further investigation.^[^ ^22^ ^]^ Subsequent studies demonstrated that WED not only promoted platelet generation but also facilitated the recovery of leukocyte counts in thrombocytopenic mice.^[^ ^23^ ^]^ However, the mechanisms underlying its effects on leukocyte recovery are not fully understood. This study aimed to explore the therapeutic potential of WED in neutropenia and elucidate its mechanisms of action using a combination of approaches, including the Gene Expression Omnibus (GEO) database, RNA sequencing, and network pharmacology. Our findings provide novel insights and strategies for the treatment of neutropenia.

## 2 Results

### 2.1 WED Induces Neutrophil Differentiation and Enhances Bactericidal Activity In Vitro

**Figure 1:**
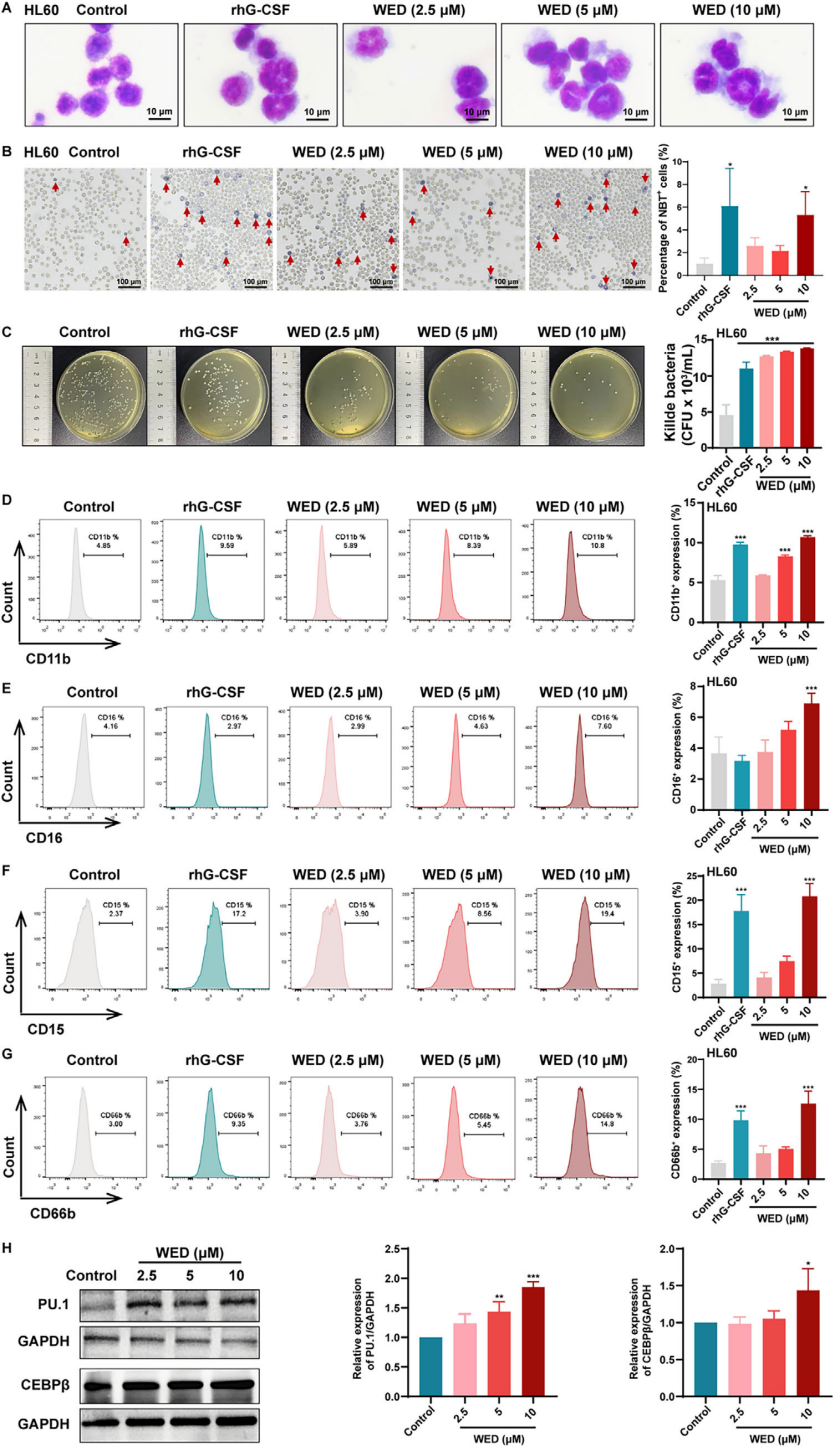
Effects of WED on neutrophil differentiation and bactericidal activity of HL60 cells. A) HL60 cells are treated with WED (2.5, 5, and 10 µm) for 5 days. Cell morphology was assessed using Giemsa staining. B) HL60 cells were treated with WED (2.5, 5, and 10 µm) for 5 days and incubated with NBT (1 mg mL^−1^). Red arrows indicated NBT‐positive (NBT⁺) cells. Quantification of NBT⁺ cells. The percentage of NBT⁺ cells was calculated as the number of NBT⁺ cells divided by the total cell count per field (*n *= 3). C) Evaluation of bactericidal activity of WED (2.5, 5, and 10 µm) on *S. aureus*. Histogram represents the killed bacteria of WED (2.5, 5, and 10 µm) on *S. Aureus* (*n* = 3). D) Effects of WED (2.5, 5, and 10 µm) on CD11b expression of HL60 cells (*n* = 3). E) Effects of WED (2.5, 5, and 10 µm) on CD16 expression of HL60 cells (*n* = 3). F) Effects of WED (2.5, 5, and 10 µm) on CD15 expression of HL60 cells (*n* = 3). G) Effects of WED (2.5, 5, and 10 µm) on CD66b expression of HL60 cells (*n* = 3). H) Effects of WED (2.5, 5, and 10 µm) on the expression of CEBPβ and PU.1 after 5 days of treatment of HL60 cells. Histograms represent the expression of CEBPβ and PU.1 of each group (*n* = 3). Data are presented as mean ± SD from at least three independent experiments. Statistical significance was determined using one‐way ANOVA followed by Tukey's post hoc test. ^*^ *p* < 0.05, ^**^ *p* < 0.01, ^***^ *p* < 0.001, versus control.

Inducing neutrophil differentiation is a promising therapeutic strategy for treating neutropenia. HL60 and NB4 cells are well‐established in vitro models for studying neutrophil differentiation. To establish safe and effective therapeutic concentrations, we first evaluated the cytotoxicity of WED using the Cell Counting Kit‐8 (CCK‐8) and Lactate Dehydrogenase (LDH) assays. CCK‐8 data revealed that WED treatment (2.5, 5, 10, and 20 µm) inhibited the proliferative capacity of HL60 and NB4 cells (Figure S1A,B, Supporting Information). However, LDH assays indicated no significant cytotoxicity at concentrations of 2.5, 5, and 10 µm WED (Figure S1C,D, Supporting Information). Therefore, these concentrations were selected for further investigation. Reduced cell proliferation is often associated with increased differentiation.^[^ ^24^ ^]^ Consequently, we evaluated the effects of WED on the differentiation of HL60 cells. Giemsa staining revealed that WED induced granulocyte differentiation, characterised by a reduced nuclear‐to‐cytoplasmic ratio and increased nuclear division (**Figure** **1**A). Functional evaluation using nitroblue tetrazolium (NBT) reduction and bacterial killing assays demonstrated a significant increase in NBT‐positive cells following WED treatment (Figure 1B), as well as a dose‐dependent enhancement of bactericidal activity that exceeded the effects of rhG‐CSF, the positive control (Figure 1C). To clarify whether WED itself exhibits bactericidal activity, we incubated WED with *S. aureus* and plated the mixture on nutrient agar to assess colony formation. No significant difference in colony numbers was observed between the control and WED groups (Figure S2, Supporting Information), indicating that WED does not possess direct bactericidal activity. These findings suggest that WED enhances neutrophil function, thereby indirectly boosting bactericidal activity. Furthermore, flow cytometry analysis confirmed that WED significantly upregulated the expression of human neutrophil surface markers CD11b, CD16, CD15, and CD66b in HL60 cells (Figure 1D–G), and similar results were obtained with NB4 cells (Figure S3A–G, Supporting Information). Additionally, haematopoietic stem and progenitor cells (HSPCs) were isolated from healthy mice and cultured with SCF (50 ng mL^−1^), IL‐3 (50 ng mL^−1^), or G‐CSF (50 ng mL^−1^) to induce differentiation into neutrophils. These cells were then divided into three groups: control, rhG‐CSF, and WED. After treatment with rhG‐CSF (50 ng mL^−1^) or WED (10 µm), the expression of neutrophil differentiation markers CD11b and Ly6G in mouse HSPCs was analysed by flow cytometry on days 1, 3, and 5. On day 1, only mild differences were observed among the control, rhG‐CSF, and WED groups; however, as differentiation progressed, both rhG‐CSF and WED markedly enhanced CD11b and Ly6G expression on days 3 and 5, with levels significantly higher than those in the control group (Figure S4A, Supporting Information). On day 5, cell morphology was further examined by Giemsa staining, and neutrophil function was evaluated using the NBT reduction assay. WED treatment increased nuclear segmentation (Figure S4B, Supporting Information) and elevated the proportion of NBT‐positive cells (Figure S4C, Supporting Information). These results demonstrate that WED exerts a time‐dependent effect on neutrophil differentiation, acting primarily during the intermediate‐to‐late stages of HSPC maturation to promote both differentiation and functional maturation. Moreover, we further investigated the effect of WED on cell proliferation during mouse primary neutrophil differentiation. The results showed that on day 3 of differentiation, WED (2.5–40 µm) inhibited cell proliferation, and on day 5, WED (1.25–40 µm) exhibited a similar inhibitory effect (Figure S4D, Supporting Information). This reduced proliferation is consistent with the notion that differentiation is often accompanied by decreased cell division, supporting WED's role in promoting neutrophil differentiation. In addition to its differentiation‐promoting effects, we next evaluated whether WED induces excessive oxidative stress or causes cellular injury during neutrophil maturation. Primary mouse HSPCs were treated with WED (2.5, 5, and 10 µm), and assays for mitochondrial membrane potential (JC‐1 assay) and DNA damage (γ‐H2AX expression) were performed. WED treatment significantly reduced γ‐H2AX expression while preserving mitochondrial membrane potential, as indicated by increased JC‐1 aggregation (Figure S4E,F, Supporting Information). These results demonstrate that WED does not trigger oxidative or genotoxic stress; rather, it maintains cellular integrity while enhancing the oxidative bactericidal capacity of differentiating neutrophils. Transcription factors PU.1 and CEBPβ co‐regulate immune‐related genes like CD16 and CD11b, playing a critical role in neutrophil differentiation.^[^ ^25^, ^26^, ^27^ ^]^ Western blot results showed that WED treatment effectively induced the expression of PU.1 and CEBPβ proteins in HL60 cells (Figure 1H and Figure S5, Supporting Information). Collectively, these findings suggest that WED has the potential to promote neutrophil differentiation and to enhance neutrophil bactericidal activity in vitro.

### 2.2 WED Accelerates Neutrophil Recovery in Irradiated Zebrafish

**Figure 2:**
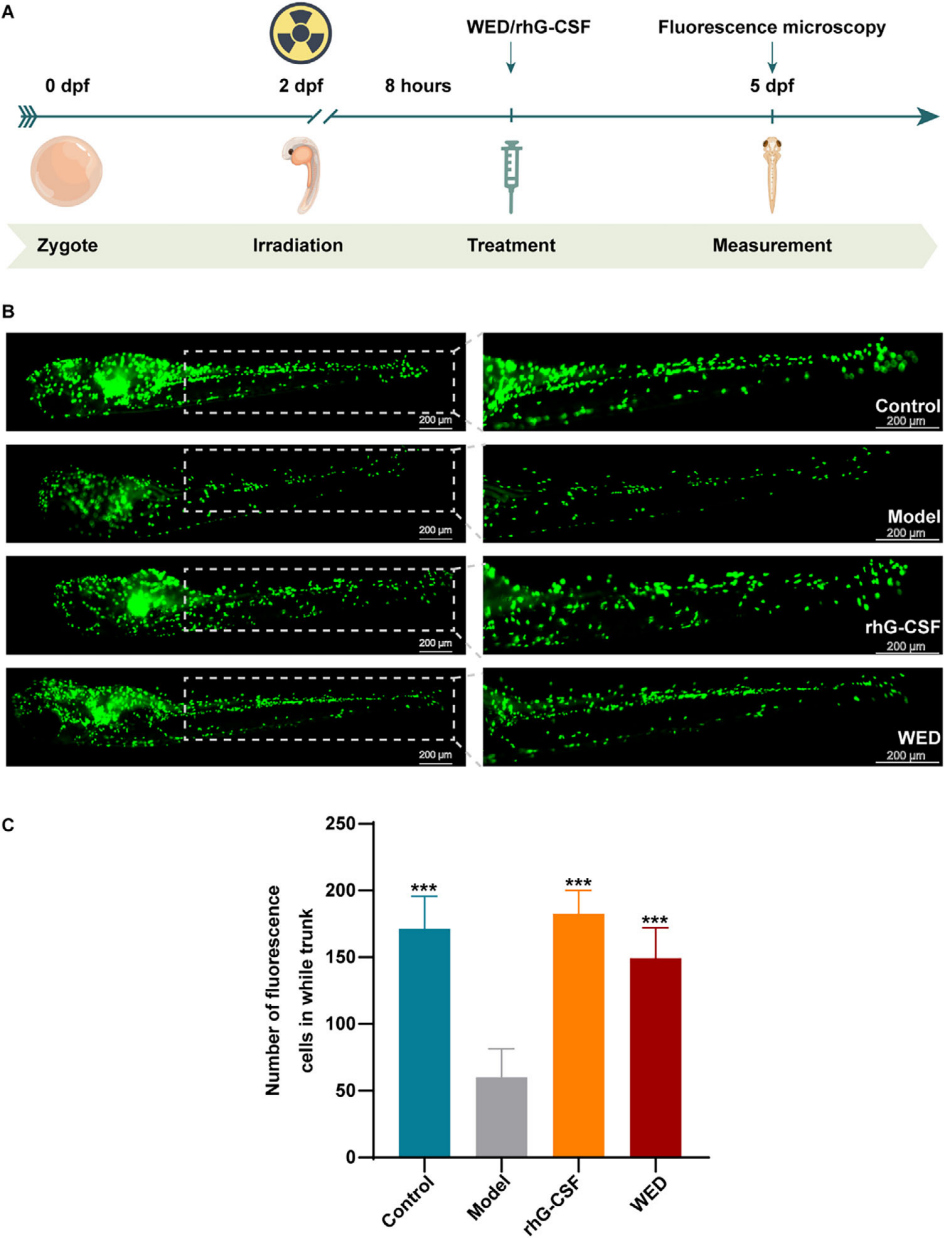
Effects of WED on neutrophil production in irradiated zebrafish. A) WED administration scheme. B) Radiation‐induced neutropenic zebrafish were treated with WED (10 µm) or rhG‐CSF (50 ng mL^−1^). The zebrafish used are transgenic Tg(mpx:eGFP) zebrafish, expressing green fluorescent protein specifically in neutrophils. C) Quantification of mpx:eGFP cells in each group (*n* = 5). Data are presented as mean ± SD from at least three independent experiments. Statistical significance was determined using one‐way ANOVA followed by Tukey's post hoc test. ^***^ *p* < 0.001, versus model.

Building upon our in vitro observations of WED‐induced neutrophil differentiation, we investigated its therapeutic potential in vivo using an irradiated zebrafish model. Tg(mpx: eGFP) transgenic zebrafish were exposed to 4 Gy of X‐ray at 2 days post‐fertilization (dpf) and subsequently treated with WED (10 µm) or rhG‐CSF. Neutrophil abundance, visualised by eGFP expression, was assessed throughout the trunk at 5 dpf (**Figure** **2**A). The number of neutrophils in the irradiated group was significantly lower compared to the control group. Notably, both WED and rhG‐CSF treatment significantly elevated neutrophil counts compared to the model group at 5 dpf (Figure 2B,C and Figure S6, Supporting Information). These findings suggest that WED accelerates neutrophil recovery in vivo in an irradiated zebrafish model.

### 2.3 WED Promotes Neutrophil Production and Enhances Bactericidal Activity in Neutropenic Mice

**Figure 3:**
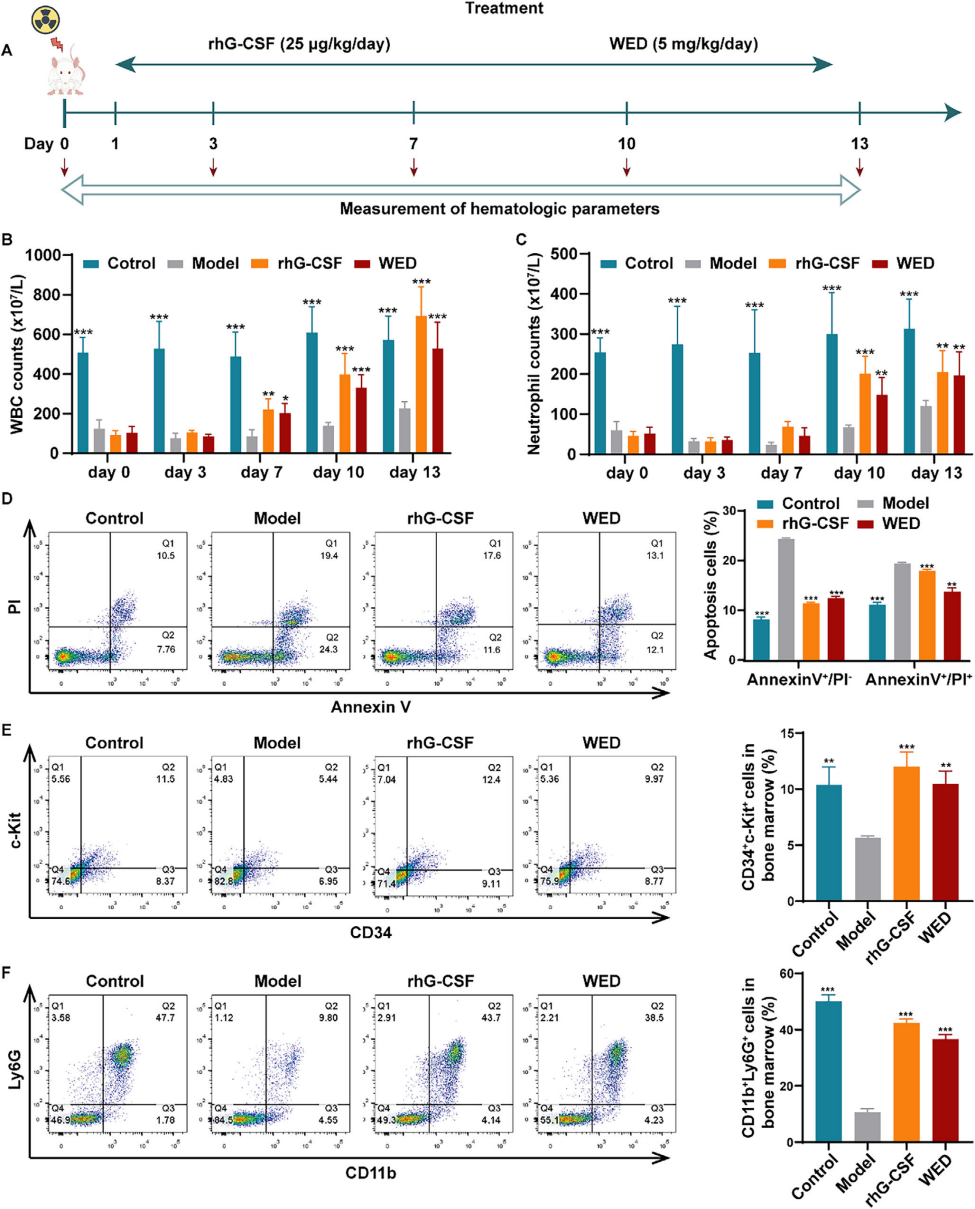
Therapeutic effects of WED on neutropenia induced by 4 Gy X‐ray irradiation in mice. A) Schematic representation of the experimental design. Mice with radiation‐induced neutropenia were treated with WED. B,C) Effects of WED on the number of WBC and neutrophils in neutropenic mice on days 0, 3, 7, 10, and 13 after irradiation (*n* = 8). D) Flow cytometry analysis of bone marrow cell apoptosis in each group. The histogram illustrates the proportion of early apoptotic (Annexin V^+^/PI^−^) and late apoptotic (Annexin V^+^/PI^+^) cells in each group (*n* = 3). E) Expression of CD34^+^/c‐Kit^+^ in bone marrow cells. The histogram presents the ratio of CD34^+^/c‐Kit^+^ expression in bone marrow cells from each group (*n* = 3). F) Expression of CD11b^+^/Ly6G^+^ in bone marrow cells. The histogram demonstrates the ratio of CD11b^+^/Ly6G^+^ expression in bone marrow cells from each group (*n* = 3). Data are presented as mean ± SD from at least three independent experiments. Statistical significance was determined using one‐way ANOVA followed by Tukey's post hoc test. ^*^ *p* < 0.05, ^**^ *p* < 0.01, ^***^ *p* < 0.001, versus model.

We further established a neutropenic mouse model using 4 Gy X‐ray irradiation and evaluated the effect of WED (5 mg kg^−1^) on neutrophil recovery and bactericidal activity (**Figure** **3**A). WBC counts in all irradiated mice reached a nadir between days 0 and 3 (Figure 3B). However, WBC numbers in the WED and rhG‐CSF groups significantly increased on days 7–13 compared to the model group (Figure 3B). Neutrophil recovery commenced on day 7, with a marked increase observed by day 10 and 13 (Figure 3C). These findings suggest that WED promotes the restoration of leukocyte levels, particularly neutrophils, following irradiation.

Bone marrow, the primary haematopoietic organ responsible for blood cell production, is particularly susceptible to radiation‐induced damage. Therefore, we used flow cytometry to assess apoptosis in mouse bone marrow cells. Our data revealed that WED treatment significantly reduced radiation‐induced apoptosis in bone marrow cells (Figure 3D). Additionally, WED treatment significantly increased the population of bone marrow CD34^+^c‐Kit^+^ hematopoietic stem cells and CD11b^+^Ly6G^+^ neutrophils compared with the model group (Figure 3E,F). These observations indicated that the differentiation of haematopoietic precursors toward the myeloid granulocyte lineage. We also isolated HSPCs from the bone marrow of irradiated mice. After 5 days of intervention, we analysed the expression of CD11b and Ly6G. Both WED (10 µm) and rhG‐CSF (50 ng mL^−1^) significantly promoted the expression of CD11b and Ly6G of HSPCs (Figure S7A, Supporting Information), confirming that WED facilitates neutrophil differentiation from HSPCs derived from irradiated mice. Giemsa staining showed that WED treatment significantly induced the formation of multi‐lobed, band‐like, or horse‐shoe‐shaped nuclei (Figure S7B, Supporting Information), which are typical morphological features of neutrophils. The NBT reduction assay revealed that WED significantly increased the number of NBT‐positive cells of HSPCs (Figure S7C, Supporting Information), indicating that WED‐treated mouse primary neutrophils were capable of performing essential immune functions.

We used immunohistochemistry (IHC) to detect Caspase‐3, a key enzyme involved in the execution phase of apoptosis, in the bone marrow. Radiation significantly increased Caspase‐3 expression, whereas WED treatment effectively suppressed this increase (Figure S8A, Supporting Information), suggesting that WED reduces radiation‐induced apoptosis in bone marrow cells. IHC analysis was performed to validate Ki67 and Ly6G expression in animal organ tissues. IHC staining also revealed higher Ki67 expression in thymus and bone marrow sections from WED and rhG‐CSF‐treated groups compared to the model group (Figure S8B,C, Supporting Information), indicating that WED promotes cell proliferation in these tissues. Moreover, IHC analysis of bone marrow demonstrated a significant increase in Ly6G expression in the WED and rhG‐CSF‐treated groups (Figure S8D, Supporting Information), suggesting enhanced neutrophil production. Considering the critical role of neutrophils as effectors in the innate immune response against bacterial infections, we assessed the bactericidal activity of neutrophils in vivo. Mice were infected with 1 × 10^6^ colony‐forming units (CFU) of *S. aureus* and euthanised 16 h later to quantify bacterial growth in the peritoneal cavity. Mice treated with WED exhibited significantly reduced *S. aureus* growth compared to both model mice and those treated with rhG‐CSF (Figure S8E, Supporting Information). We also measured ROS levels, finding no significant effect of WED treatment on ROS levels (Figure S9A, Supporting Information), indicating that WED did not regulate radiation‐induced oxidative stress. γ‐H2AX, a well‐known biomarker for DNA damage induced by ionising radiation,^[^ ^28^ ^]^ was assessed in bone marrow cells by flow cytometry. Radiation significantly increased γ‐H2AX expression, while WED treatment notably reduced the radiation‐induced elevation of γ‐H2AX (Figure S9B, Supporting Information), indicating that WED alleviates DNA damage induced by radiation

Given the pronounced hematopoietic effects of WED observed in the radiation‐induced neutropenia model, we next sought to determine whether these actions were radiation‐specific or reflected a broader hematopoietic regulatory potential. To this end, we conducted additional studies in normal mice and in a chemotherapy‐induced neutropenic model. In normal mice, continuous administration of WED (5 mg kg^−1^) for 13 days transiently increased total WBC and neutrophil counts on days 7 and 10 compared with the control group, with both parameters returning to baseline by day 13 (Figure S10A,B, Supporting Information). This pattern was comparable to that observed in the rhG‐CSF group (Figure S10A,B, Supporting Information). Flow cytometry further showed that WED significantly increased the proportions of bone marrow CD34⁺c‐Kit⁺ hematopoietic stem/progenitor cells and CD11b⁺Ly6G⁺ neutrophils (Figure S10C,D, Supporting Information), suggesting that WED enhanced physiological hematopoiesis and neutrophil differentiation even under non‐stressed conditions. Moreover, WED reduced basal apoptosis in bone marrow cells but did not significantly alter ROS levels or γ‐H2AX expression (Figure S10E–G, Supporting Information), indicating that WED promoted hematopoietic homeostasis without inducing oxidative or genotoxic stress.

To further verify the generalizability of WED's effects beyond radiation injury, we established a carboplatin‐induced neutropenic mouse model, which closely mimics the myelosuppressive toxicity observed in clinical chemotherapy.^[^ ^29^ ^]^ Consistent with the radiation model, carboplatin markedly decreased WBC and neutrophil counts, whereas WED (5 mg kg^−1^) and rhG‐CSF (25 µg kg^−1^) treatment significantly accelerated hematologic recovery from day 7 onward, restoring normal levels by day 13 (Figure S10A,B, Supporting Information). Flow cytometry confirmed that WED increased CD34⁺c‐Kit⁺ cells and CD11b⁺Ly6G⁺ neutrophil populations, while reducing carboplatin‐induced apoptosis, ROS accumulation, and γ‐H2AX expression in bone marrow cells (Figure S10C–G, Supporting Information). Collectively, these findings demonstrate that WED exerts consistent hematopoietic and pro‐neutrophilic activities across multiple conditions—including physiological, radiation‐induced, and chemotherapy‐induced myelosuppression. Rather than acting through a radiation‐specific mechanism, WED functions as a broad‐spectrum hematopoietic modulator that enhances myeloid differentiation, preserves bone marrow integrity, and accelerates systemic hematologic recovery.

### 2.4 Analysis of Gene Expression Profiles in Neutropenia Patients

**Figure 4:**
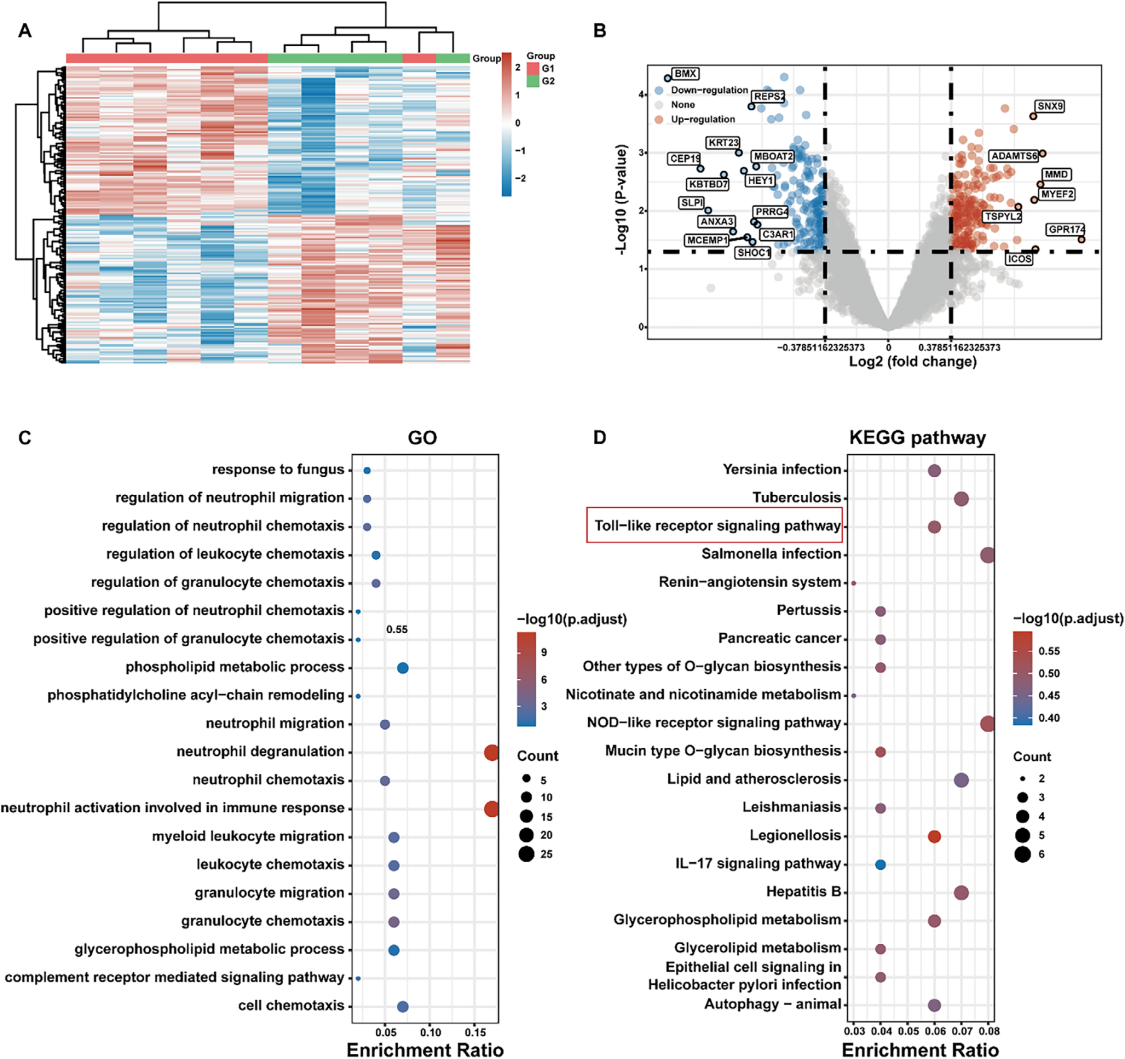
Gene expression analysis in human peripheral blood granulocytes from the GEO database. A) Hierarchical clustering analysis of DEGs between neutropenic patients (G1) and healthy controls (G2). B) Volcano plot depicting DEGs. Red dots indicate upregulated genes, grey dots correspond to genes without significant differences, and blue dots represent downregulated genes. C) GO enrichment analysis. D) KEGG enrichment analysis.

To identify potential mechanisms underlying neutropenia, we analysed gene expression profiles of human peripheral blood granulocytes from patients with neutropenia and healthy controls. The GEO database was queried using the search term “neutropenia,” and the GSE108894 dataset was selected, which comprised five control samples and seven samples from patients with neutropenia. Following normalisation, batch correction, and log transformation of gene expression data, differentially expressed genes (DEGs) were identified using a fold change (FC) ≥ 1.3 and an adjusted *p‐value* < 0.05. A total of 351 DEGs were identified, including 187 upregulated and 164 downregulated genes. Volcano and heatmap visualisations were used to depict the DEGs (**Figure** **4**A,B).

To further explore potential therapeutic pathways, we performed Gene Ontology (GO) and Kyoto Encyclopedia of Genes and Genomes (KEGG) pathway enrichment analyses. GO enrichment analysis revealed significant enrichment of DEGs in biological processes associated with the immune system, including neutrophil activation involved in immune response, neutrophil degranulation and granulocyte chemotaxis (Figure 4C). KEGG pathway enrichment analysis indicated that the Toll‐like signaling pathway, IL‐17 signaling pathway, and NOD‐like receptor signaling pathway may represent promising therapeutic targets for neutropenia (Figure 4D).

### 2.5 RNA Sequencing Unveils the Transcriptional Landscape Regulated by WED

**Figure 5:**
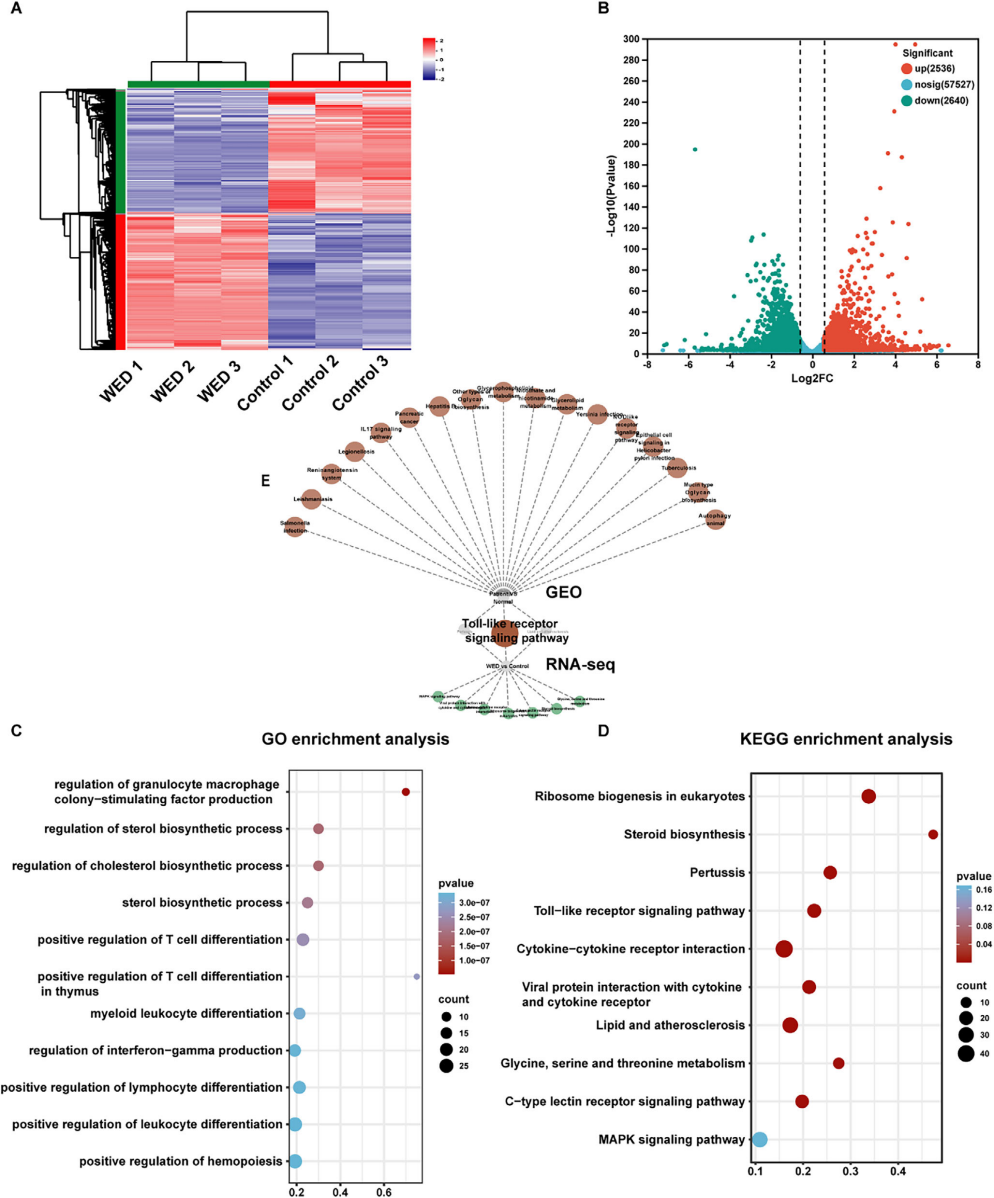
RNA sequencing analysis. A) Gene expression changes between the control group and WED‐treated groups of HL60 cells. B) Volcano plot indicating DEGs regulated by WED. C) GO enrichment analysis. D) KEGG enrichment analysis. E) Visualization of the commonly enriched signaling pathways identified in DEGs from both the GEO database and RNA sequencing analysis.

To elucidate the molecular mechanism by which WED promoted neutrophil differentiation, we performed RNA sequencing of HL60 cells to generate a comprehensive transcriptional profile. Differential expression analysis identified 5176 DEGs between the control and WED‐treated groups (2536 upregulated and 2640 downregulated) (**Figure** **5**A,B). Hierarchical clustering analysis of the DEGs revealed distinct expression patterns between the control and WED‐treated groups, indicating robust clustering and high sample reproducibility (Figure 5A). A volcano plot was generated to visualize the differential expression patterns (Figure 5B).

To gain insights into the functional roles of WED‐regulated DEGs, we performed GO and KEGG pathway enrichment analyses. GO analysis revealed significant enrichment of DEGs in biological processes associated with the immune and haematopoietic systems, including the regulation of granulocyte macrophage colony‐stimulating factor production, positive regulation of T cell differentiation in thymus, positive regulation of haematopoiesis, and myeloid leukocyte differentiation (Figure 5C). These findings suggested that WED exerts a positive regulatory effect on haematopoiesis, particularly leukocyte differentiation. KEGG analysis identified pathways that were significantly regulated by WED, such as ribosome biogenesis in eukaryotes, Toll‐like receptor signaling pathway, cytokine‐cytokine receptor interaction, and MAPK signaling pathway (Figure 5D). These pathways may be involved in the molecular mechanisms underlying WED‐mediated neutrophil differentiation.

Additionally, we compared the pathway enrichment results from the GEO database neutropenia dataset (GSE108894) with the DEGs identified in our RNA sequencing analysis. Network visualization using Cytoscape software (version 3.9.1) revealed a significant overlap in the Toll‐like receptor signaling pathway (Figure 5E). Given the crucial role of the Toll‐like receptor signaling pathway in the immune system, particularly in pathogen defence and immune homeostasis, we hypothesize that WED promotes neutrophil differentiation primarily through this pathway.

### 2.6 Network Pharmacology Combined with Transcriptome Sequencing Predicts the Targets of WED in the Treatment of Neutropenia

**Figure 6:**
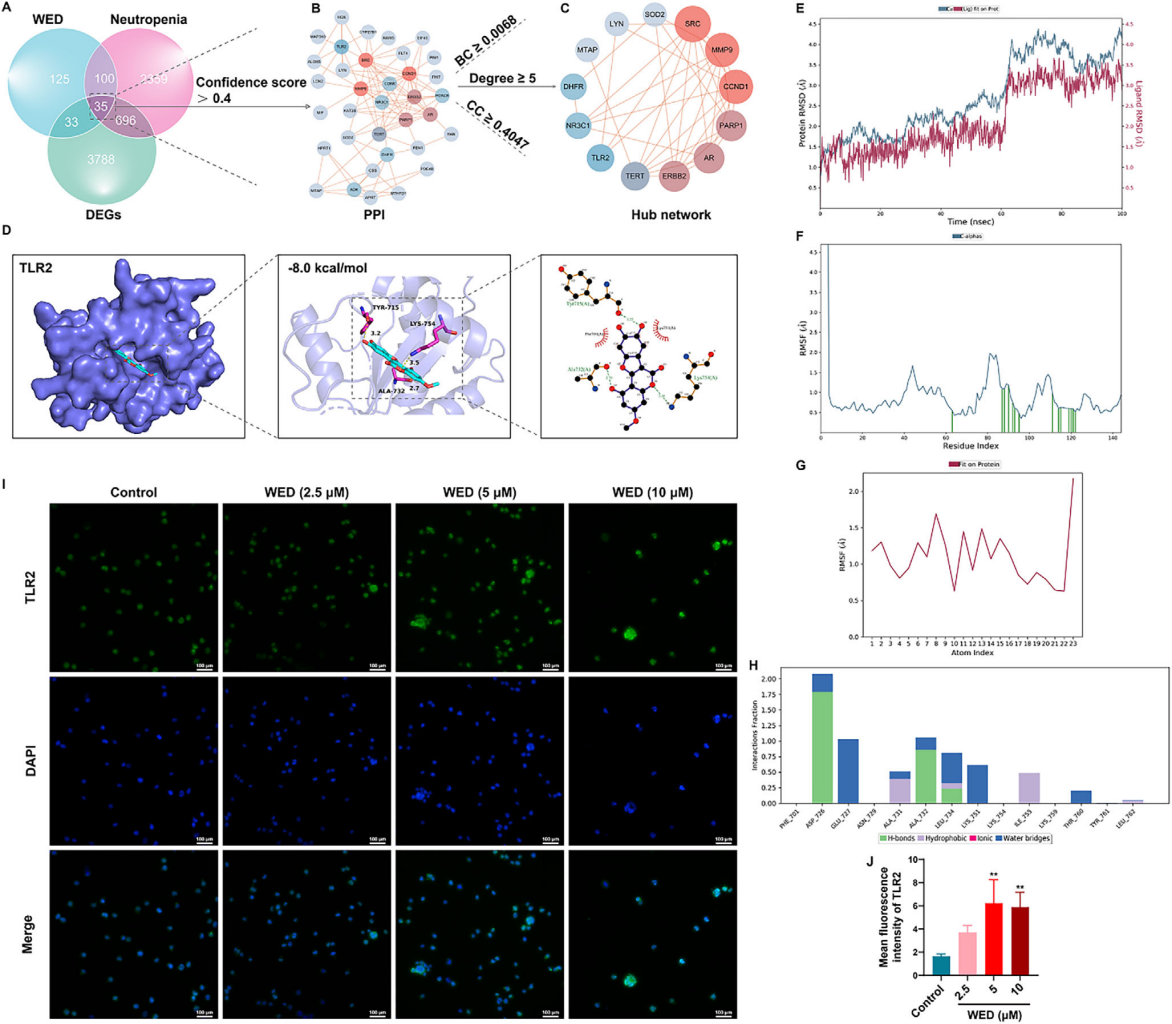
Predicted targets of WED in neutropenia treatment. A) The Venn diagram illustrating the 35 common targets identified among potential targets of WED targets, neutropenia‐related targets, and DEGs from RNA sequencing analysis. B) PPI network of shared targets. C) Potential core target of WED treatment. D) Molecular docking reveals the interaction between WED and TLR2. E) Time‐dependent RMSD plots of TLR2 Cα atoms and the ligand WED during a 100‐ns molecular dynamics simulation. F) RMSF profile of TLR2 residues. G) RMSF profile of WED heavy atoms. H) Interaction fraction analysis showing hydrogen bonds, hydrophobic, ionic, and water‐bridge interactions between WED and key residues of TLR2. I) Immunofluorescence analysis of TLR2 expression in HL60 cells after treatment with WED (2.5, 5, and 10 µm) for 5 days. J) Quantification of mean fluorescence intensity of TLR2 (*n* = 3). Data are presented as mean ± SD from at least three independent experiments. Statistical significance was determined using one‐way ANOVA followed by Tukey's post hoc test. ^**^ *p* < 0.01, versus control.

To identify potential therapeutic targets for WED in neutropenia, we integrated network pharmacology with RNA sequencing data. Using established databases, we compiled a list of 293 potential WED and 3190 neutropenia‐associated targets. After intersecting these datasets with the 4552 DEGs identified through RNA sequencing after removing duplicates, we found 35 common dysregulated targets (**Figure** **6**A). To prioritize these potential targets, the 35 identified genes were uploaded to the STRING 12.0 database for protein‐protein interaction (PPI) network analysis. A high‐confidence interaction network was constructed using stringent criteria (composite score > 0.4), resulting in a network with 35 nodes and 99 edges (Figure 6B). Network topology analysis identified 13 core targets, including TLR2, SRC, and MMP9, with topological eigenvalues (degree, betweenness, and closeness) exceeding the median were identified (Figure 6C).

Our findings, coupled with those of previous studies, suggest that WED may promote neutrophil differentiation primarily through the Toll‐like receptor signaling pathway. Notably, TLR2 emerged as one of the core targets identified through the intersectional target topology analysis. TLR2 activation modulates adaptive immune responses by promoting immune cell activation and proliferation, ultimately enhancing the host's specific defence against pathogens.^[^ ^12^ ^]^ Therefore, TLR2 is a strong candidate for mediating WED‐induced neutrophil differentiation.

### 2.7 WED Promotes Neutrophil Differentiation by Directly Binding to TLR2

**Figure 7:**
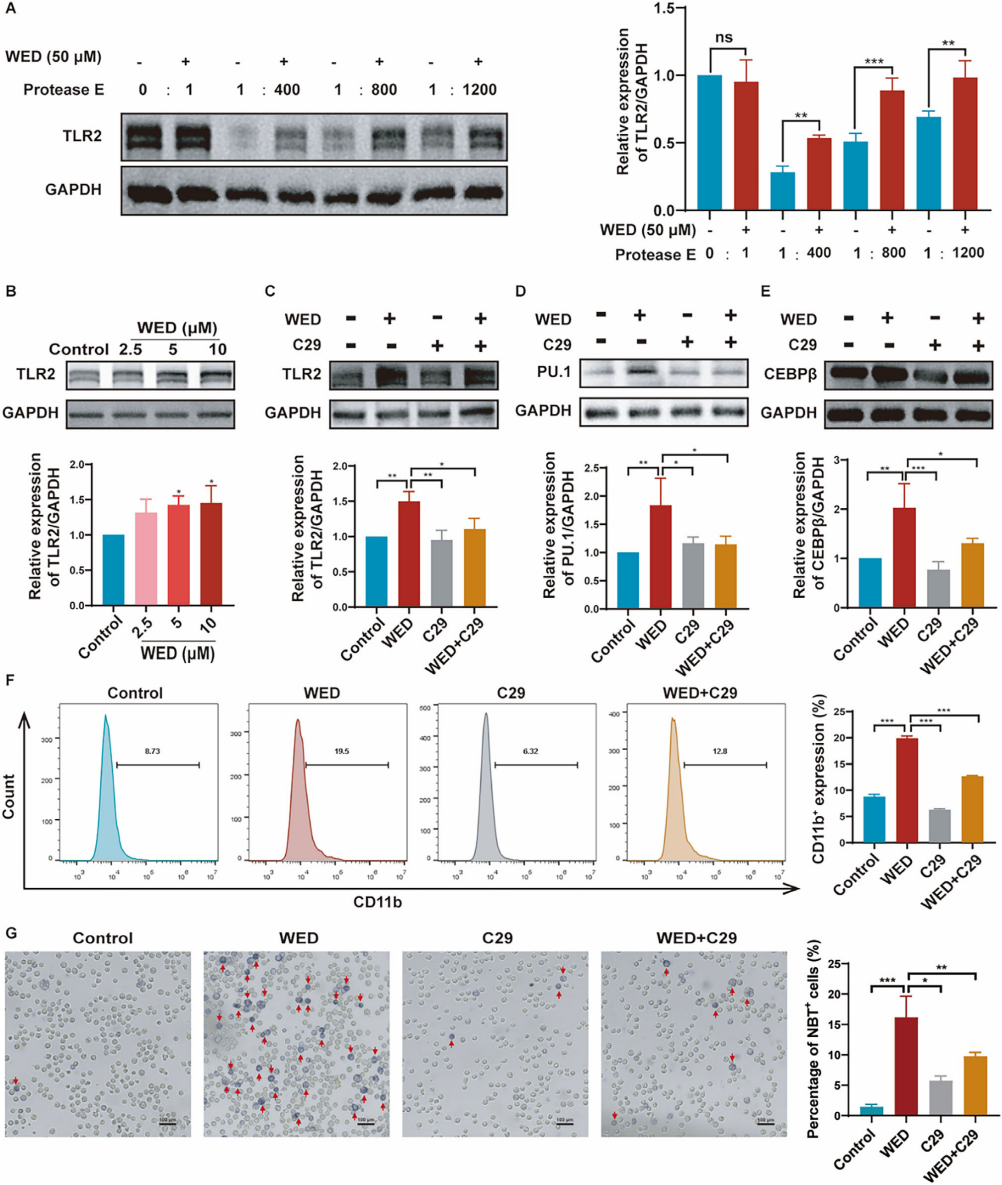
WED directly interacts with TLR2 to regulate neutrophil differentiation. A) DARTS assay demonstrating the direct binding between WED and TLR2 in HL60 cells. B–E) Western blot analysis of TLR2, PU.1, and CEBPβ expression in HL60 cells treated with WED (2.5, 5, and 10 µm), or WED (10 µm), C29 (50 µm), and WED (10 µm) + C29 (50 µm) for 5 days. F) Flow cytometry analysis of CD11b expression in HL60 cells treated with WED and C29 for 5 days. G) Detection of NBT reducing activity after treatment with WED and C29 for 5 days in HL60 cells. Quantification of NBT⁺ cells. The percentage of NBT⁺ cells was calculated as the number of NBT⁺ cells divided by the total cell count per field (*n* = 3). Data are presented as mean ± SD from at least three independent experiments. Statistical significance was determined using one‐way ANOVA followed by Tukey's post hoc test. ^*^p < 0.05, ^**^p < 0.01, ^***^p < 0.001, versus the corresponding control groups.

To validate TLR2 as a target for WED‐mediated neutrophil differentiation, we first performed molecular docking. WED formed a well‐defined binding pose within the TLR2 pocket, engaging multiple stabilizing interactions; notably, Tyr715 established a hydrogen bond with the ligand. Under these interactions, the computed binding energy was −8.0 kcal mol^−1^, consistent with a thermodynamically favorable complex (Figure 6D). To further assess complex stability in an explicit solvent environment, molecular dynamics simulations were conducted. The Cα‐RMSD of TLR2 displayed an initial equilibration phase followed by stabilization at ≈3.8 Å, while the ligand RMSD converged to ≈3.3 Å (Figure 6E), indicating only modest deviations from the docked conformation and supporting a stable, equilibrated binding mode over the production trajectory. RMSF analysis showed generally low fluctuations for residues lining the binding site and for ligand heavy atoms, consistent with restricted local flexibility at the protein–ligand interface (Figure 6F,G). Hydrogen‐bond occupancy analysis revealed persistent contacts throughout the simulation; in particular, Asp726 formed hydrogen bonds with high frequency (occupancy ≈80% and ≈98% for two donor–acceptor pairs) (Figure 6H), underscoring its key anchoring role. Additional transient hydrogen bonds and water‐mediated bridges, together with hydrophobic contacts in the pocket, further contributed to complex stabilization (Figure 6H). Collectively, the molecular dynamics simulations results indicate that WED and TLR2 undergo limited conformational drift relative to the docked pose and evolve toward a more stable bound ensemble during the simulation. Immunofluorescence staining and western blot analysis revealed that WED treatment significantly enhanced TLR2 expression in HL60 cells compared to the control group (Figure 6I,J, **Figures** **7**B and S11, Supporting Information). Consistent with the docking and molecular dynamics simulations results, the Drug Affinity Responsive Target Stability (DARTS) assay further revealed that WED treatment protected TLR2 from protease degradation in HL60 cells (Figure 7A and Figure S11, Supporting Information), demonstrating that WED directly binds to TLR2. To investigate the role of TLR2 in WED‐mediated neutrophil differentiation, we used the classical TLR2 inhibitor C29. C29 treatment significantly inhibited WED‐induced TLR2 expression in HL60 cells (Figure 7C and Figure S11, Supporting Information). Similarly, C29 treatment also inhibited the expression of PU.1 and CEBPβ (Figure 7D,E and Figure S11, Supporting Information), CD11b expression (Figure 7F), and NBT reduction activity (Figure 7G) induced by WED in HL60 cells. Additionally, we investigated the effects of combined treatment with C29 and WED on normal mouse‐derived HSPCs. The results showed that C29 treatment significantly inhibited the expression of CD11b and Ly6G induced by WED (Figure S12A, Supporting Information), indicating that TLR2 inhibition suppresses WED‐induced neutrophil differentiation in primary mouse HSPCs. To further confirm the role of TLR2 in neutrophil differentiation, we treated normal mouse‐derived HSPCs with Pam3CSK4, a well‐established TLR2 agonist. Pam3CSK4 treatment significantly promoted the expression of CD11b and Ly6G (Figure S12B, Supporting Information), suggesting that TLR2 plays a pivotal role in promoting neutrophil differentiation from primary mouse HSPCs. To provide direct genetic evidence, we further employed small interfering RNA (siRNA)–mediated TLR2 knockdown in primary mouse HSPCs. Three siRNAs targeting TLR2 were designed and validated by western blot analysis, among which siRNA‐1 and siRNA‐2 achieved significant suppression of TLR2 protein expression (Figure S13A, Supporting Information). Functional assays showed that WED markedly upregulated CD11b and Ly6G expression in the WED + negative control (NC) group compared with NC alone, whereas TLR2 knockdown (siRNA‐1 or siRNA‐2) reduced basal differentiation (Figure S13B,C, Supporting Information). Importantly, co‐treatment with WED + TLR2 siRNA significantly diminished the WED‐induced increase in CD11b and Ly6G expression (Figure S13B,C, Supporting Information), confirming that TLR2 was indispensable for WED's pro‐differentiation effect. Taken together, these results demonstrate that WED targets TLR2 to promote neutrophil differentiation and enhance their bactericidal activity.

### 2.8 The MAPK Signaling Pathway Is Activated During WED‐Promoted Neutrophil Differentiation and Maturation

**Figure 8:**
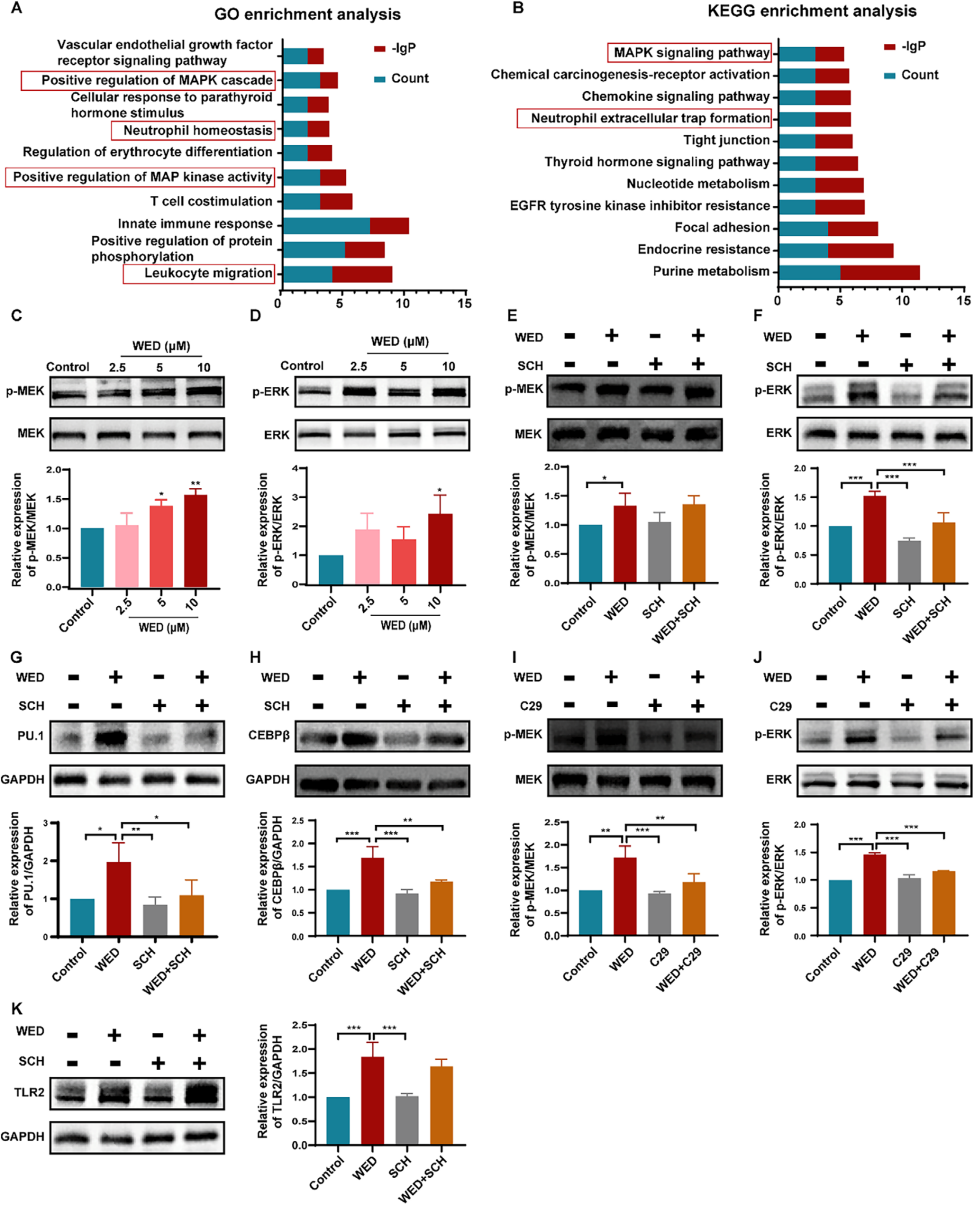
WED targets TLR2 and activates the MEK/ERK pathway to promote neutrophil differentiation. A,B) GO and KEGG enrichment analysis of the 35 common targets. C–H) Western blot analysis of p‐MEK, p‐ERK, PU.1, and CEBPβ expression in HL60 cells treated with WED (2.5, 5, and 10 µm), or WED (10 µm), SCH (2.5 µm), and WED (10 µm) + SCH (2.5 µm) for 5 days (*n* = 3). I,J) Western blot analysis of MEK and ERK phosphorylation following treatment with WED (10 µm) and C29 (50 µm) for 5 days in HL60 cells (*n* = 3). K) Western blot analysis of TLR2 expression after treatment with WED (10 µm) and SCH (2.5 µm) for 5 days in HL60 cells (*n* = 3). Data are presented as mean ± SD from at least three independent experiments. Statistical significance was determined using one‐way ANOVA followed by Tukey's post hoc test. ^*^ *p* < 0.05, ^**^ *p* < 0.01, ^***^ *p* < 0.001, versus the corresponding control groups.

To gain a deeper mechanistic understanding of WED's therapeutic effects on neutropenia, we combined network pharmacology with RNA sequencing and performed GO and KEGG functional enrichment analyses on the 35 common targets identified previously. These analyses yielded results consistent with the RNA sequencing findings. GO enrichment analysis revealed that these targets were involved in diverse cellular processes related to the immune and haematopoietic systems, including leukocyte migration, innate immune response, positive regulation of MAP kinase activity, neutrophil homeostasis, and positive regulation of the MAPK cascade (**Figure** **8**A). These findings suggest that WED actively modulates the innate immune system, particularly leukocyte function, and primarily regulates the MAPK pathway. Furthermore, KEGG analysis indicated that WED primarily regulates neutrophil extracellular trap formation and the MAPK signaling pathway (Figure 8B), highlighting its potential to induce neutrophil differentiation and enhance their bactericidal capacity. These results further supported the involvement of the MAPK pathway in WED‐induced neutrophil differentiation.

Previous reports have emphasized the critical role of ERK1/2 in neutrophil production and bactericidal function by regulating the activation of myeloid transcription factors such as PU.1 and CEBPβ.^[^ ^24^ ^]^ Our western blot data demonstrated that WED significantly promoted MEK and ERK phosphorylation in HL60 cells, suggesting that it activates the MEK/ERK signaling pathway (Figure 8C,D and Figure S14, Supporting Information). To further investigate the role of ERK in WED‐induced neutrophil differentiation, we used the highly selective ERK inhibitor SCH772984 (SCH). SCH effectively inhibited WED‐induced ERK activation (Figure 8E,F and Figure S14, Supporting Information) and suppressed the expression of PU.1 and CEBPβ induced by WED in HL60 cells (Figure 8G,H and Figure S14). This was further confirmed by the downregulation of CD11b expression and the reduction in the number of NBT‐positive cells in HL60 cells (Figure S15A,B, Supporting Information). To further explore the role of the MAPK signaling pathway in neutrophil differentiation, we treated mouse‐derived HSPCs with PMA, which activates protein kinase C (PKC) and indirectly activates the MAPK signaling pathway.^[^ ^30^, ^31^ ^]^ PMA treatment significantly promoted the expression of CD11b and Ly6G (Figure S12B, Supporting Information), confirming the key role of the MAPK signaling pathway neutrophil differentiation. These results collectively suggest that WED can induce neutrophil differentiation through activation of the MAPK signaling pathway. We also investigated the roles of other pathways, including PI3K/Akt, JAK/STATs, and receptor tyrosine kinase (RTK) signaling pathways, which are commonly associated with immune cell function and neutrophil differentiation.^[^ ^32^, ^33^, ^34^, ^35^, ^36^ ^]^ Using inhibitors specific to these pathways—LY294002 (PI3K inhibitor), Ruxolitinib (JAK1/2 inhibitor), and Dovitinib (multi‐targeted RTK inhibitor for FLT3, c‐Kit, CSF‐1R, FGFR1/FGFR3, VEGFR1/VEGFR2/VEGFR3 and PDGFRα/PDGFRβ)—we found that none of these inhibitors blocked WED‐induced CD11b expression in HL60 cells (Figure S16, Supporting Information). These results suggest that the PI3K/Akt, JAK2/STAT, and RTK pathways do not mediate the effects of WED on neutrophil differentiation.

Since TLR2 is known to activate MAPK and other signaling cascades upon ligand binding,^[^ ^37^, ^38^ ^]^ we further explored the relationship between TLR2 and MAPK during WED‐induced neutrophil differentiation. Treatment with the TLR2 inhibitor C29 suppressed WED‐induced MEK/ERK activation in HL60 cells (Figure 8I,J and Figure S14, Supporting Information). Consistently, western blot analysis further demonstrated that TLR2 knockdown by siRNA‐1 in primary mouse HSPCs not only reduced TLR2 protein expression but also markedly attenuated MEK and ERK phosphorylation, while co‐treatment with WED failed to restore this activation (Figure S13D‐F and S17, Supporting Information). These findings, together with the pharmacological inhibition results, provide compelling genetic evidence that WED promotes neutrophil differentiation through the TLR2–MEK/ERK signaling axis. Additionally, the enhancement of WED‐induced TLR2 expression was not inhibited by the ERK inhibitor (SCH) in HL60 cells (Figure 8K and Figure S14, Supporting Information), suggesting that WED activates TLR2/MEK/ERK signaling pathway to promote neutrophil differentiation. We also examined the expression of TLR2, MEK, ERK, PU.1, and CEBPβ in the bone marrow cells of neutropenic mice induced by irradiation. Compared to the model group, WED treatment promoted the TLR2 expression, MEK and ERK phosphorylation, and enhanced the expression of PU.1 and CEBPβ (Figures S18 and S19, Supporting Information), further supporting that WED activates the TLR2–MEK/ERK axis to promote neutrophil differentiation and restore hematopoietic function.

## 3 Discussion

Neutropenia, defined as a decrease in the absolute neutrophil count in peripheral blood, is a common and severe complication in cancer patients, especially those undergoing chemotherapy and radiotherapy.^[^ ^39^ ^]^ Neutrophils are critical components of the immune system, primarily responsible for the defence against bacterial and fungal infections.^[^ ^3^ ^]^ Neutropenia significantly weakens immune function, increasing the risk of infections and potentially leading to life‐threatening complications, particularly in cancer patients.^[^ ^40^ ^]^ Chemotherapy and radiotherapy often cause severe bone marrow suppression, resulting in a dramatic decrease in neutrophil levels, which increases susceptibility to infections and complicates the treatment of the primary disease, thereby raising the risk of treatment‐related mortality.^[^ ^41^, ^42^ ^]^ The aetiology of neutropenia is complex and multifactorial. In addition to chemotherapy‐ and radiotherapy‐induced bone marrow suppression, neutropenia can result from immune disorders, infections, and genetic conditions.^[^ ^43^, ^44^, ^45^, ^46^ ^]^ Common acquired causes include drug reactions, autoimmune diseases, viral infections (e.g., Epstein‐Barr virus, HIV), and splenic disorders.^[^ ^43^, ^44^, ^45^ ^]^ Genetic conditions, such as congenital neutropenia, can result in sustained neutrophil reductions and recurrent severe infections.^[^ ^46^ ^]^ Despite the diverse causes, all these conditions share a common feature: they impair the immune system, making patients highly susceptible to infections.^[^ ^43^, ^44^, ^45^, ^46^ ^]^ Current treatment strategies, such as G‐CSF, can stimulate neutrophil production to some extent and alleviate bone marrow suppression‐induced neutropenia. However, these treatments are not effective for all patients and are often accompanied by side effects such as fatigue, dizziness, and recurrent infections.^[^ ^47^ ^]^ Additionally, G‐CSF is often less effective for patients with congenital neutropenia or those with neutropenia due to immune destruction or genetic mutations.^[^ ^43^, ^46^, ^48^ ^]^ Consequently, existing therapies are insufficient to meet the clinical needs, specifically targeting the underlying mechanisms of neutropenia. New therapies should not only promote neutrophil production but also modulate immune responses and the bone marrow microenvironment, overcoming the limitations of current treatments.

In response to this clinical challenge, we focused on WED, a natural coumarin, as a potential therapeutic agent for neutropenia. In vitro experiments demonstrated that WED significantly upregulated the expression of PU.1 and CEBPβ, two key transcription factors involved in neutrophil differentiation in HL60 cells. This was further substantiated by the increased expression of mature neutrophil markers such as CD11b, CD16, CD15, and CD66b, as well as enhanced neutrophil function against bacteria in HL60 and NB4 cells. In normal HSPCs derived from healthy mice, WED also promoted neutrophil differentiation and enhanced neutrophil immune functions. This was evidenced by the increased expression of CD11b and Ly6G (mouse neutrophil differentiation markers), as well as an increase in the number of multi‐lobed nuclei and NBT‐positive cells following WED treatment.

In vivo, the hematologic and functional findings observed across multiple models collectively demonstrate that WED acts as a physiological modulator of hematopoiesi. In the radiation‐induced neutropenia model, WED promoted the recovery of peripheral white blood cell and neutrophil counts, reduced bone marrow apoptosis, and restored hematopoietic balance, thereby facilitating the coordinated regeneration of the myeloid compartment. The parallel improvement in bacterial clearance further suggests that WED restores both the quantity and quality of neutrophils, supporting the re‐establishment of effective innate immune defence. The consistency of these findings in normal and chemotherapy‐induced myelosuppression models reinforces the concept that WED possesses a broad hematopoietic regulatory capacity rather than a radiation‐specific effect. In normal mice, transient increases in leukocyte and neutrophil counts without signs of oxidative or genotoxic stress indicate that WED enhances physiological hematopoiesis within homeostatic limits. In the carboplatin model, WED accelerated hematologic recovery and preserved bone marrow integrity, mitigating the myelosuppressive effects commonly associated with cytotoxic chemotherapy. Such reproducibility across different models highlights that WED promotes hematopoietic resilience and supports marrow regeneration under both physiological and injury conditions. Taken together, these results suggest that WED acts through a multifaceted hematopoietic regulatory mechanism that integrates progenitor cell preservation, lineage differentiation, and immune restoration. Its balanced hematopoietic stimulation, without inducing oxidative stress or genomic instability, underscores its translational potential as a safe and effective therapeutic candidate for the management of therapy‐related neutropenia and other forms of bone marrow suppression.

To comprehensively elucidate the mechanism by which WED treats neutropenia, we employed a combined approach using the GEO database, RNA sequencing, and network pharmacology. By leveraging publicly available data from the GEO database of patients with neutropenia and healthy controls, we identified potential therapeutic pathways for neutropenia. Network pharmacology facilitated the prediction of potential WED targets and associated signaling pathways, offering insights into its anti‐neutropenia effects. RNA sequencing enabled us to investigate the effects of WED on gene expression in neutrophils, further revealing the molecular mechanisms governing its effects on neutrophil function and differentiation. Differential gene enrichment analysis of GEO and RNA sequencing data highlighted the Toll‐like receptor signaling pathway as a potentially critical player in WED‐induced neutrophil differentiation. Subsequent network pharmacology, coupled with transcriptome sequencing analysis, yielded a PPI network that identified TLR2 as a potential core target. The direct interaction between WED and TLR2 was subsequently validated through a series of computational and biochemical assays. Molecular docking revealed that WED fits into the TLR2 binding pocket through multiple stabilizing interactions, with Tyr715 forming a hydrogen bond that contributed to a calculated binding energy of −8.0 kcal mol^−1^. Further molecular dynamics simulations confirmed the stability of this complex, showing that both the receptor and ligand exhibited limited RMSD fluctuation, while key residues such as Asp726 maintained persistent hydrogen‐bonding interactions throughout the trajectory—highlighting the conformational stability and high affinity of the WED–TLR2 complex. Consistent with these computational findings, DARTS assays demonstrated that WED protected TLR2 from proteolytic degradation, providing direct biochemical evidence for physical binding between WED and TLR2. Beyond structural validation, functional and genetic evidence further substantiated the essential role of TLR2 in mediating WED's effects. Pharmacological blockade of TLR2 with C29 or genetic silencing using siRNA in primary mouse HSPCs both markedly attenuated WED‐induced neutrophil differentiation. These results demonstrate that TLR2 is indispensable for the pro‐differentiation and pro‐hematopoietic activity of WED. Together, these computational (molecular docking and molecular dynamics simulations), biochemical (DARTS), pharmacological (C29 inhibitor), and genetic (siRNA knockdown) lines of evidence establish TLR2 as the direct molecular target through which WED promotes neutrophil differentiation. Although the present study provides strong in vitro and in silico support for the TLR2‐dependent mechanism, we acknowledge the limitation of not using TLR2‐knockout mice. Future studies employing TLR2‐deficient models will be necessary to provide in vivo genetic validation of this mechanism and to further substantiate the role of TLR2 in mediating WED's hematopoietic effects.

TLR2 is a pivotal immune receptor involved in the innate immune response.^[^ ^49^ ^]^ It is a type I transmembrane protein predominantly located on the surface of immune cells such as macrophages, dendritic cells, and neutrophils.^[^ ^50^ ^]^ Its structure consists of an extracellular N‐terminus, a transmembrane domain, and an intracellular C‐terminus. TLR2 recognizes various PAMPs such as bacterial lipopolysaccharides, lipoproteins, and lipopeptides, thereby initiating immune responses through the activation of downstream signaling pathways.^[^ ^51^ ^]^ Upon binding PAMPs, TLR2 recruits adaptor proteins, triggering downstream signal transduction pathways such as NF‐κB, MAPKs, and interferon regulatory factors.^[^ ^52^ ^]^ This activation cascade leads to the production of inflammatory factors, induction of apoptosis, production of antimicrobial proteins, and the activation and proliferation of immune cells.^[^ ^52^ ^]^ Previous studies have demonstrated that TLR2 agonists, such as GSK3277329 (a truncated analogue of Pam2CSK4), administered through daily injections for one week, significantly increased circulating neutrophil counts in healthy monkeys beyond normal levels. Furthermore, when the injections were continued for two weeks, the neutrophil levels in chemotherapy‐treated monkeys (with cyclophosphamide and Cytoxan) were effectively restored. These findings, in conjunction with our results, provide preclinical evidence that TLR2 activation promotes neutrophil production and suggest that this approach may be offer a promising therapeutic strategy for managing chemotherapy‐induced neutropenia.^[^ ^12^ ^]^ TLR2 can enhance neutrophil functions such as pathogen killing and cytokine release, thus strengthening the local immune response.^[^ ^53^, ^54^ ^]^ We also observed that the TLR2 inhibitor C29 significantly blocked the WED‐induced increase in the number of NBT‐positive cells, suggesting that TLR2 not only mediates WED‐induced neutrophil differentiation but also contributes to their enhanced immune function.

Functional enrichment analysis of the common targets identified by network pharmacology and RNA sequencing revealed that WED primarily influences the innate immune response, neutrophil homeostasis, and positive regulation of the MAPK cascade. ERK1/2, a member of the MAPK family, is known to regulate neutrophil generation through the activation of myeloid transcription factors like PU.1 and CEBPβ.^[^ ^55^ ^]^ Therefore, we further investigated the role of the MAPK pathway in WED‐induced neutrophil differentiation. Western blot demonstrated that WED promoted MEK/ERK phosphorylation, indicating the activation of the MAPK pathway. Using an ERK inhibitor, we confirmed the role of MAPK signalling in neutrophil differentiation. Inhibition of the MEK/ERK pathway significantly reduced WED‐induced neutrophil differentiation and maturation, as evidenced by the downregulation of CD11b expression and a decrease in the NBT‐positive cell population. Notably, treatment with both the TLR inhibitor C29 and the ERK inhibitor SCH further suppressed the WED‐induced expression of PU.1 and CEBPβ. These findings collectively suggest that the upregulation of key neutrophil differentiation transcription factors (PU.1 and CEBPβ) by WED necessitates the involvement of both TLR2 and the MEK/ERK pathway.

**Figure 9:**
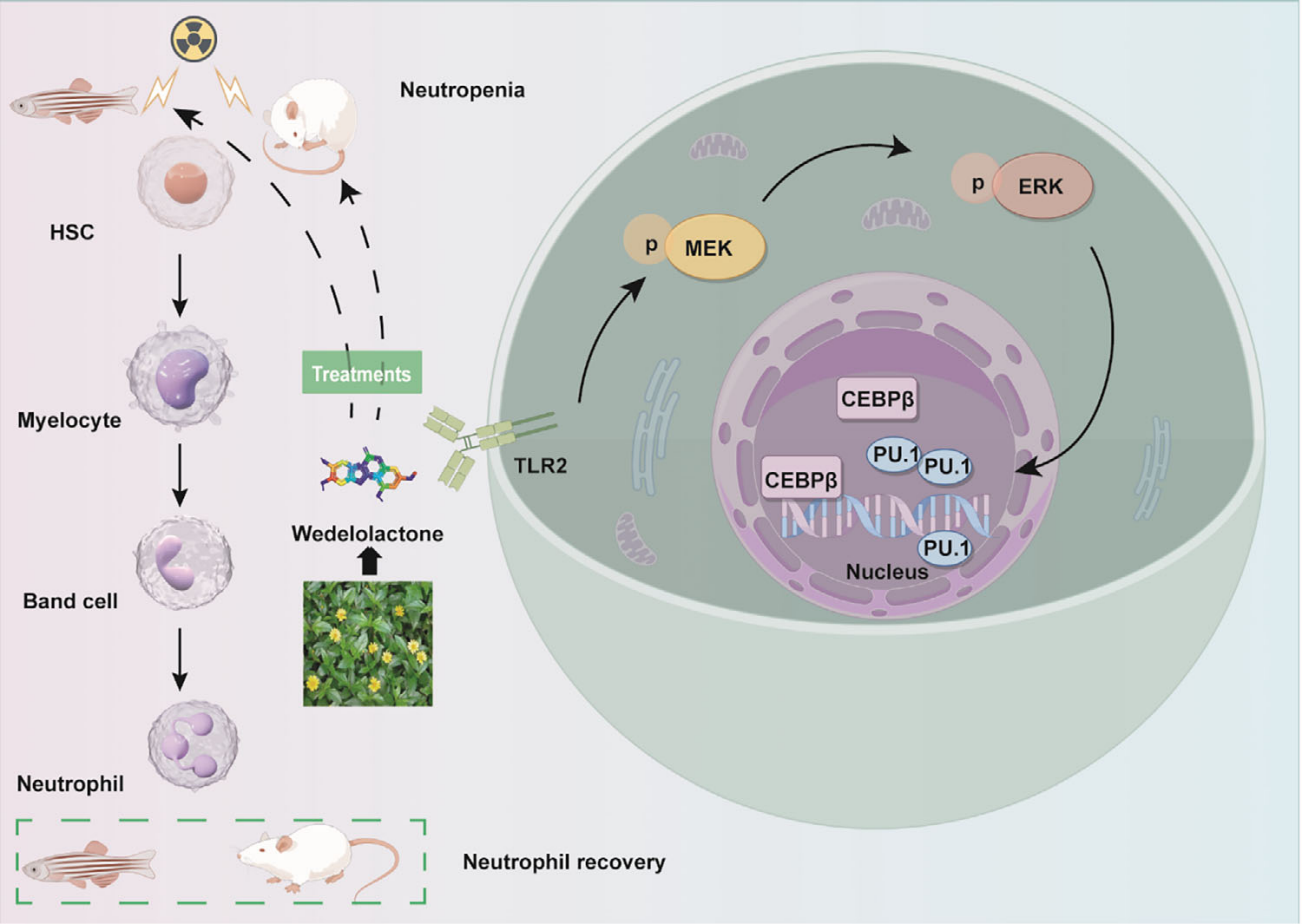
Molecular mechanism of WED‐mediated neutrophil differentiation via TLR2/MEK/ERK pathway activation. The mechanism diagram was created using Figdraw.

To further elucidate the hierarchy within the signaling cascade, we investigated the potential of TLR2 as an upstream regulator of the MEK/ERK pathway in WED‐induced neutrophil differentiation. Treatment with the C29 or TLR2 siRNA significantly suppressed WED‐induced phosphorylation of MEK/ERK. Conversely, the introduction of SCH did not affect TLR2 expression, indicating that WED targets TLR2 to activate the downstream MAPK pathway. This, in turn, regulates the activation of myeloid transcription factors PU.1 and CEBPβ, ultimately facilitating neutrophil differentiation (**Figure** **9**).

Despite the convergent in vitro, in vivo, and in silico evidence supporting a TLR2–MEK/ERK–PU.1/CEBPβ axis, our study still has limitations with regard to the exact level at which WED operates within the hematopoietic hierarchy. Most of our functional assays were performed in heterogeneous hematopoietic cell populations and in whole bone marrow settings, in which neutrophil committed progenitors, upstream HSPCs, and niche or accessory cells coexist and can all respond to TLR2 activation. In addition, TLR2 is expressed across multiple myeloid differentiation stages rather than being restricted to a single neutrophil precursor subset. Therefore, although our data indicate that WED enhances granulocytic maturation and neutrophil function, they do not allow us to definitively separate direct, cell‐intrinsic effects on neutrophil committed progenitors from indirect regulation via more primitive HSPCs or the bone marrow microenvironment. In this context, WED is best viewed as a hematopoietic modulator that preferentially supports neutrophil lineage output and functional recovery, rather than as an exclusively neutrophil‐specific and purely direct regulator. Future work employing single‐cell transcriptomics, in vivo lineage tracing, and conditional TLR2 ablation in defined progenitor compartments will be required to dissect the relative contributions of direct versus indirect actions in greater detail.

In summary, building on previous research, this study further elucidated WED's ability to induce neutrophil differentiation and enhance neutrophil bactericidal functions in both in vivo and in vitro models. Using a multifaceted approach that integrated GEO database data, RNA sequencing, and network pharmacology, we identified potential targets of WED and elucidated downstream pathways. Subsequent experimental validation demonstrated that WED targeted TLR2, activated the downstream MAPK pathway, and modulated the activation of myeloid transcription factors PU.1 and CEBPβ. In conclusion, our findings underscore the potential of TLR2 agonists in mitigating radiotherapy‐ or chemotherapy‐induced neutropenia and highlight WED as a novel TLR2 agonist with promising therapeutic potential for treating neutropenia. This comprehensive approach provides a valuable foundation for further development of WED as a potential clinical treatment.

## 4 Experimental Section

### Chemicals

WED, with a purity exceeding 99.66%, as determined by high‐performance liquid chromatography (HPLC), was procured from Chengdu Pusi Biotechnology Co., Ltd. (Chengdu, China), and dissolved according to the manufacturer's instructions.

### Cell Culture

The HL60 and NB4 cell lines were obtained from the American Type Culture Collection (Bethesda, MD, USA). The cells were cultured in RPMI‐1640 medium supplemented with 10% foetal bovine serum (FBS) and 1% penicillin/streptomycin.

### Isolation and Culture of Mouse HSPCs

Irradiated and normal mice were euthanized under anesthesia, and femurs and tibias were carefully dissected to expose the bone marrow cavities. Bone marrow cells were flushed out using a 1 mL syringe into a 50 mL centrifuge tube, with repeated rinsing until the bones appeared completely white. HSPCs were isolated from the bone marrow of normal or irradiated mice using the EasySep Mouse Haematopoietic Progenitor Cell Isolation Kit (STEMCELL Technologies, Vancouver, Canada). The isolated cells were adjusted to a density of 3 × 10^5^ cells mL^−1^ and seeded into 24‐well plates. Cultures were maintained in StemSpan serum‐free expansion medium (STEMCELL Technologies, Vancouver, Canada) supplemented with 1% penicillin/streptomycin and specific growth factors, including SCF (50 ng mL^−1^; Peprotech) and IL‐3 (50 ng mL^−1^; Peprotech).^[^ ^56^ ^]^ On the third day of culture, either rhG‐CSF (50 ng mL^−1^), WED (10 µm), C29 (50 µm), Pam3CSK4 (100 ng mL^−1^), or PMA (10 ng mL^−1^) was administered as an intervention. Relevant parameters were assessed on day 5 following treatment.

### Cell Proliferation Assay

The proliferation of NB4, HL60, and mouse HSPCs cells was assessed using the CCK‐8 assay and LDH assay kit (Beyotime, Shanghai, China), following the manufacturer's protocols. Briefly, HL60 and NB4 cells were seeded at a density of 2 × 10⁴ cells mL^−1^, and mouse HSPCs were plated at 3 × 10⁵ cells mL^−1^ in 96‐well plates. The cells were exposed to varying concentrations of WED (2.5, 5, 10, and 20 µm) for 1, 3, and 5 days. Untreated cells served as the control group. Following treatment, the CCK‐8 or LDH assay working solution was added, and the absorbance was measured according to the instructions.

### Giemsa Staining

The NB4 and HL60 cells were exposed to or without WED (2.5, 5, and 10 µm) for 5 days, with rhG‐CSF (50 ng mL^−1^) used as the positive control group.^[^ ^37^ ^]^ HL60 and NB4 cells were seeded at a density of 2 × 10⁴ cells mL^−1^ in 12‐well plates. Mouse HSPCs were treated with or without WED (10 µm) or rhG‐CSF (50 ng mL^−1^) and plated at a density of 3 × 10⁵ cells mL^−1^. Following treatments, cells were harvested, fixed, and incubated with Giemsa solution (Solarbio, Beijing, China) for a duration of 5 min. Photomicrographs were captured using a light microscope (NIKON, Japan).

### Quantification of Cells Surface Markers

Following the 5‐day treatment with WED (2.5, 5, and 10 µm) or rhG‐CSF, HL60 and NB4 cells were collected and incubated with APC anti‐human CD11b, FITC anti‐human CD16, FITC anti‐human CD15 (4A Biotech, Beijing, China), or FITC anti‐human CD66b (Biolegend, San Diego, CA, USA) antibodies for 30 min, shielded from light. HL60 and NB4 cells were seeded at a density of 2 × 10⁴ cells mL^−1^ in 12‐well plates. Similarly, HSPCs were collected and incubated with FITC‐conjugated anti‐human/mouse CD11b (4A Biotech, Beijing, China) and PE‐conjugated anti‐mouse Ly6G (Elabscience, Wuhan, China) antibodies for 30 min. Mouse HSPCs were plated at a density of 3 × 10⁵ cells mL^−1^ in 12‐well plates. After staining, cells were analysed using flow cytometry (BD Biosciences, San Jose, USA).

### NBT Reduction Assay

HL60, NB4 cells, or HSPCs were incubated at 37 °C in phosphate‐buffered saline (PBS) containing NBT (1 mg mL^−1^) and 12‐O‐tetradecanoylphorbol‐13‐acetate (PMA, 1 µg mL^−1^) (Beyotime, Shanghai, China) for 45 min. HL60 and NB4 cells were seeded at a density of 2 × 10⁴ cells mL^−1^, while mouse HSPCs were plated at 3 × 10⁵ cells mL^−1^. After incubation, cells containing precipitated formazan particles were immobilised using methanol. Photomicrographs of the cells were captured using a light microscope (NIKON, Japan). The percentage of NBT⁺ cells was calculated as the number of NBT⁺ cells divided by the total cell count per field.

### Bacteria Killing

Cells subjected to various treatments were harvested on the fifth day and washed three times with PBS. HL60 and NB4 cells were seeded at a density of 2 × 10⁴ cells mL^−1^ and counted prior to co‐incubation to ensure accurate cell numbers. Subsequently, 1 × 10⁵ cells were co‐incubated with *S. aureus* at a bacteria‐to‐cell ratio of 10:1 for 90 min at 37 °C, in triplicate. After incubation, the suspensions were serially diluted, plated onto nutrient agar, and incubated overnight at 37 °C. Colony counts were recorded the following day. The number of killed bacteria was calculated using the formula: Killed bacteria = No. _blank –_ No. _Tested_.

### Animals

The transgenic zebrafish strain Tg(mpx:eGFP) was provided by the public platform of zebrafish technology of Southwest Medical University (Luzhou, China) and housed in a circulating system at 28 ± 0.5 °C with a 14‐h light and 10‐h dark cycle. Kunming (KM) mice (Liaoning Changsheng Biological Co., Ltd., Liaoning, China), aged 8–10 weeks, were fed a standard diet and maintained under a 12‐h light/12‐h dark cycle. The laboratory animal ethics committee of the Southwest Medical University (Luzhou, China) approved and monitored experimental procedures in accordance with the guidelines (License No. 20230904‐052).

### Establishment and Treatment of Neutropenic Zebrafish Model

At 2 dpf, transgenic Tg(mpx:eGFP) larvae were exposed to X‐rays (4 Gy) as previously described.^[^ ^47^ ^]^ After irradiation, the zebrafish were transferred to 6‐well plates and treated with WED (10 µm) and rhG‐CSF. The non‐irradiated zebrafish group served as the control. Fluorescently labelled neutrophils were imaged and counted using a stereo fluorescence microscope (Leica, Wetzlar, Germany).

### Establishment and Treatment of Radiation‐Induced Neutropenic Mouse Model

The mice were divided randomly into four groups: control group, neutropenia model group, rhG‐CSF‐positive group (25 µg kg^−1^), and WED (5 mg kg^−1^) group. Except for the control group, the other groups were exposed to 4 Gy X‐ray irradiation to induce the neutropenia. After irradiation, the control and model groups received daily intraperitoneal injections of normal saline. The G‐CSF and WED groups received intraperitoneal injections of G‐CSF or WED for 13 consecutive days. Blood samples (40 µL) were collected from the ocular venous plexus on days 0, 3, 7, and 10 and analyzed using a haematology analyser (Sysmex XT‐2000iV, Kobe, Japan).

### Establishment and Treatment of Carboplatin‐Induced Neutropenic Mouse Model

The mice were randomly divided into two major cohorts: a normal cohort (*n* = 24) and a chemotherapy‐induced neutropenic cohort (*n* = 24). For the normal cohort, mice were randomly assigned into three groups: control group, rhG‐CSF‐positive group (25 µg kg^−1^), and WED (5 mg kg^−1^) group. Each group received daily intraperitoneal injections with normal saline, rhG‐CSF, or WED administered for 13 consecutive days. For the chemotherapy cohort, mice were intraperitoneally injected with carboplatin (50 mg kg^−1^) to induce neutropenia. After model establishment, animals were randomly divided into three groups: model group, rhG‐CSF‐positive group (25 µg kg^−1^), and WED (5 mg kg^−1^) group. Drug administration was performed once daily for 13 consecutive days. Peripheral blood samples (40 µL) were collected from the ocular venous plexus on days 0, 3, 7, 10, and 13, and leukocyte and neutrophil counts were measured using a haematology analyzer (Sysmex XT‐2000iV, Kobe, Japan).

### Flow Cytometry Analysis of Bone Marrow

Bone marrow cells were harvested and prepared according to standard protocols.^[^ ^57^ ^]^ For haematopoietic stem and progenitor analysis, cells were labelled with FITC‐conjugated anti‐CD34 (4A Biotech, Beijing, China) and PE‐conjugated anti‐CD117 (4A Biotech, Beijing, China). For neutrophil analysis, cells were labelled with FITC‐conjugated anti‐CD11b (4A Biotech) and APC‐conjugated anti‐Ly6G/Ly6c (4A Biotech). All antibody incubations were carried out for 20 min. For apoptosis analysis, Annexin V‐FITC and propidium iodide (PI) (4A Biotech, Beijing, China) were added to the cell suspension and incubated for 15 min. For ROS analysis, the ROS Assay Kit (Beyotime, Shanghai, China) was added, and cells were incubated for 20 min. For DNA damage analysis, cells were fixed with 70% ethanol and incubated with FITC‐conjugated anti‐phospho‐H2A.X (BioLegend, San Diego, CA, USA) for 40 min at room temperature in the dark. Mitochondrial membrane potential was evaluated using the Mitochondrial Membrane Potential Assay Kit JC‐1 (Beyotime, Shanghai, China) following the manufacturer's instructions. After staining, cells were analysed by flow cytometry (BD Biosciences, San Jose, USA).

### Bacteria Killing in Vivo

Bacteria were resuspended in sterile saline before injection, and adjusted to the desired concentration. To induce peritonitis, mice were inoculated with 1 × 10^6^ CFU of *S. aureus* via intraperitoneal injection. After 16 h, the mice were euthanised, and *S. aureus* was isolated from the peritoneal cavity by washing it with 6 mL of sterile saline. *S. aureus* cells were resuspended in sterile saline, diluted, and plated onto nutrient agar. The plates were incubated overnight at 37 °C, and the number of bacterial colonies was counted the following day.

### IHC Analysis

Mice were euthanized, and their femur and thymus were isolated and fixed in 10% paraformaldehyde. The femurs were decalcified in a decalcification solution for over a month. Organs were subsequently embedded in paraffin, sectioned, and incubated with antibodies against Caspase‐3 (Abone marrowart, Shanghai, China), Ki67 (Proteintech, IL, USA), and Ly6G (Abone marrowart, Shanghai, China). Photomicrographs were captured using an Olympus BX 51 microscope (Olympus Optical, Tokyo, Japan).

### Western Blot Analysis

For immunoblotting, the samples were loaded onto 10% SDS‐PAGE gels and then transferred to polyvinyl difluoride (PVDF) membrane (BioRad, Hercules, CA). After blocking, primary antibodies were applied and incubated overnight. The membranes were washed three times with PBST, followed by incubation with secondary antibodies at 37 °C for 1 h. Primary antibodies used included: GAPDH (Abmart, P30008), TLR2 (Abmart, TU362792S), p‐ERK (Abmart, PC3292), ERK (Abmart, T40071), p‐MEK (Cell Signalling Technology, 2338S), MEK (Cell Signalling Technology, 4694S), PU.1 (Yping Bio, YP‐Ab‐01964), and C/EBPβ (Yping Bio, YP‐53b‐00220).

### DARTS Assay

Proteins were extracted from HL60 cells and adjusted to a concentration of 5 mg mL^−1^ prior to drug treatment. Proteins were treated with WED or dimethyl sulfoxide (DMSO) for 1 h at room temperature. Subsequently, a series of protease dilutions (1:400, 1:800, and 1:1200) were added, and the samples were incubated at 40 C for 10 min. The 5× sample buffer was added, and the samples were boiled for 10 min. Western blot was used to analyse the samples and evaluate the extent of protein degradation, using GAPDH as an internal control.

### Immunofluorescence

Following stimulation with various treatments, HL60 cells were collected, fixed with 4% paraformaldehyde, and treated with 0.5% Triton X‐100 for 15 min. Cells were seeded at a density of 2 × 10⁴ cells mL^−1^ prior to treatment. Next, the cells were incubated with a primary anti‐TLR2 antibody (1:200, Abone marrowart, Shanghai, China, TU362792S) at 4 C overnight. The cells were then incubated with a FITC‐labelled goat anti‐rabbit secondary antibody (1:500; Beyotime, Shanghai, China) at room temperature for 1 h. Cells were then rinsed with PBS, restained with DAPI (100 nm; Solarbio, Beijing, China), and analysed under a fluorescence microscope.

### TLR2 siRNA Transfection in Mouse HSPCs

Mouse HSPCs were isolated and cultured as described above. For siRNA transfection, cells were seeded in 24‐well plates at a density of 3 × 10⁵ cells mL^−1^ and incubated overnight to allow cell recovery. When cell confluence reached ≈60%, transfection was performed using the siRNA‐Mate Plus reagent (Genechem Co., Ltd., Shanghai, China) according to the manufacturer's instructions. Briefly, for each well, 17 µL of Buffer and 30 pmol of siRNA were gently mixed, followed by the addition of 3 µL siRNA‐Mate Plus reagent. The mixture was pipetted gently for homogeneity and then added dropwise to the cells. The plates were gently agitated and incubated under standard conditions. After 72 h of transfection, TLR2 expression levels were analyzed by western blot to confirm knockdown efficiency. Twenty‐four hours after siRNA transfection, rhG‐CSF (50 ng mL^−1^) or WED (10 µm) was added to the cultures. Differentiation parameters were evaluated on day 5 post‐treatment. Three siRNA sequences targeting mouse TLR2 were designed and synthesized by Genechem Co., Ltd. (Shanghai, China) as follows: siRNA‐1: sense 5′‐GCCUUGACCUGUCUUUCAATT‐3′; antisense 5′‐UUGAAAGACAGGUCAAGGCTT‐3′. siRNA‐2: sense 5′‐GAGACUUUCAGUGAGAUAATT‐3′; antisense 5′‐UUAUCUCACUGAAAGUCUCTT‐3′. siRNA‐3: sense 5′‐CGAGCUGGGUAAAGUAGAATT‐3′; antisense 5′‐UUCUACUUUACCCAGCUCGTT‐3′. The NC siRNA sequence was: sense 5′‐UUCUCCGAACGUGUCACGUTT‐3′; antisense 5′‐ACGUGACACGUUCGGAGAATT‐3′.

### Identification and Functional Enrichment of Neutropenia‐Related DEGs

The GEO database (https://www.ncbi.nlm.nih.gov/geo/) was searched using the keyword “neutropenia,” and the dataset GSE108894 was selected.^[^ ^58^ ^]^ This dataset included five normal samples and seven samples from patients with neutropenia. The raw data was downloaded as MINiML files, and log2 normalisation was performed. The normalize.quantiles function in the preprocessCore package in R was used for data standardisation. Gene symbols were derived from the annotation information of the normalised data on the platform. Differentially expressed mRNAs were identified using the limma package (version 3.40.2) in R, with adjusted *p‐values* to correct for false‐positives. The ClusterProfiler package (version 3.18.0) in R was used to analyse GO functions of potential targets and enrich KEGG pathways.^[^ ^59^ ^]^

### RNA Sequencing

Total RNA was extracted using TRIzol (Invitrogen, Carlsbad, CA) following the treatment of HL60 cells with WED (10 µm) for 3 days. RNA quality was assessed using the 2100 Bioanalyzer system (Agilent Technologies, USA) and the ND‐2000 NanoDrop spectrophotometer (Nanodrop Technologies, France). RNA libraries were prepared using the TruSeq RNA Sample Preparation Kit (Illumina, San Diego, CA, USA) and sequenced on the Illumina HiSeq xten/NovaSeq 6000 platform (2 × 150‐bp) by Shanghai Metso BioMedical Biotechnology Co., Ltd. (Shanghai, China). DEGs between the control and WED‐treated groups were identified using a FC ≥ 1.5 and a *p‐value* < 0.05. These DEGs were subjected to functional enrichment analysis.

### Network Pharmacology Analysis

WED targets were retrieved from the TCMSP database (https://old.tcmsp‐e.com/index.php), the Swiss TargetPrediction database (http://swisstargetprediction.ch/), and the PharmMapper database (https://www.lilab‐ecust.cn/pharmmapper/). Neutropenia‐associated genes were obtained from the DisGeNET database (https://www.disgenet.org/) and the GeneCards database (https://www.genecards.org/). Finally, common targets were obtained by plotting drug targets, DEGs from RNA sequencing data, and disease‐related genes on a Venn diagram. The obtained common targets were imported into the STRING12.0 database (https://cn.string‐db.org/) for network topology analysis (based on a composite score > 0.4). Three network topology parameters (including “Degree,” “Betweenness Centrality,” and “Closeness Centrality”) of the common targets were calculated from the network analysis and used as the basis for scoring.^[^ ^60^ ^]^ Core targets were defined as those with topological parameters above the median.^[^ ^61^ ^]^ Interaction networks were visualized using Cytoscape software (version 3.9.1).

### Functional Enrichment Analysis

The DAVID database (https://david.ncifcrf.gov/) was used for GO functional enrichment analysis, and the Metascape database (https://metascape.org/) was used for pathway enrichment analysis. Results were visualised using the bioinformatics database (https://www.bioinformatics.com.cn/).

### Molecular Docking Validation

The PDB file format (PDB ID: 1FYW), from which the 3D arrangement of the TLR2 protein was downloaded, was obtained from the RCSB PDB database (https://www.rcsb.org/). Small‐molecule files in SDF format were retrieved from the PubChem database and modified using Open Babel software. Molecular docking was performed using AutoDock Vina to calculate the binding affinity between the receptor and ligand. An affinity ≤ ‐5.0 kcal mol^−1^ was considered indicative of a strong interaction between the receptor and ligand.^[^ ^43^ ^]^ The docking results were visualized using PyMol software.

### Molecular Dynamics Simulations

The molecular dynamics simulations were performed using the Desmond/Maestro noncommercial version 2022.1.^[^ ^62^ ^]^ The protein–ligand complex was solvated in a cubic box with TIP3P water molecules and neutralized with 0.15 m NaCl. The system was energy‐minimized and equilibrated under standard conditions before production runs. The 100 ns molecular dynamics simulation was conducted in an isothermal–isobaric ensemble at 300 K and 1 bar. Trajectories were recorded every 100 ps for analysis. Post‐simulation analyses, including root mean square deviation (RMSD), root mean square fluctuation (RMSF), and hydrogen bond evaluation, were carried out using the Simulation Interaction Diagram module in Desmond.

### Statistical Analysis

All data are presented as the mean ± standard deviation (SD) from at least three independent experiments. Data normality and variance homogeneity were examined before analysis. Differences between the two groups were evaluated using an unpaired two‐tailed Student's *t*‐test, while multiple group comparisons were performed using one‐way ANOVA followed by Tukey's post hoc test. The exact sample size (*n*) for each analysis is indicated in the figure legends. Statistical analyses were conducted using GraphPad Prism 8.0, and a *p* < 0.05 was considered statistically significant.

## Conflict of Interest

The authors declare no conflict of interest.

## Author Contributions

L.W. and Z.L. contributed equally to this work. L.W., R.L., and J.W. conceived and designed the study. L.W., Z.L., T.H., Q.L., L.Z., X.M., X.Q., and S.L. performed the experiments and collected the data. W.K., J.L., A.W., and F.H. contributed to methodology and data analysis. S.D. performed computational modeling and software analysis. S.L. contributed to visualization. C.Z., R.L., and J.W. supervised the project and revised the manuscript. L.W. and Z.L. drafted the manuscript. All authors reviewed and approved the final version of the manuscript.

## Supporting information



Supporting Information

## Data Availability

The data that support the findings of this study are available from the corresponding author upon reasonable request.
