# Supplementary material for: Wedelolactone, a Novel TLR2 Agonist, Promotes Neutrophil Differentiation and Ameliorates Neutropenia: A Multi‐Omics Approach to Unravel the Mechanism
Source: Adv Sci (Weinh). 2025 Dec 23;13(12):e09807. doi: 10.1002/advs.202509807 (PMC12948265; doi:10.1002/advs.202509807)
Supplement: Supplementary file 1 — Supporting Information [file ADVS-13-e09807-s001.docx]

Supporting Information

Wedelolactone, a Novel TLR2 Agonist, Promotes Neutrophil Differentiation and Ameliorates Neutropenia: A Multi-omics Approach to Unravel the Mechanism

Long Wang,^1^ Zhichao Li,^1^ Tianci Hu, Qinyao Li, Linwei Zhang, Xinyue Mei, Xiao Qi, Sheng Liu, Weijie Kong, Jiesi Luo, Anguo Wu, Feihong Huang, Sirui Li, Shuang Dai, Chunxiang Zhang,* Rong Li,* and Jianming Wu*

Corresponding authors:

Jianming Wu

School of Basic Medical Sciences, Southwest Medical University, Luzhou, Sichuan 646000, China

E-mail: [jianmingwu@swmu.edu.cn](mailto:jianmingwu@swmu.edu.cn)

Rong Li

Laboratory for Cardiovascular Pharmacology of Department of Pharmacology, The School of Pharmacy, Southwest Medical University, Luzhou, Sichuan 646000, China

E-mail: [lr2008@swmu.edu.cn](mailto:lr2008@swmu.edu.cn)

Chunxiang Zhang

Key Laboratory of Medical Electrophysiology, Sichuan Key Medical Laboratory of New Drug Discovery and Druggability Evaluation, Luzhou Key Laboratory of Activity Screening and Druggability Evaluation for Chinese Materia Medica, Southwest Medical University, Luzhou, Sichuan 646000, China

E-mail: [zhangchx999@163.com](mailto:zhangchx999@163.com)

Long Wang and Zhichao Li contributed equally to this work.


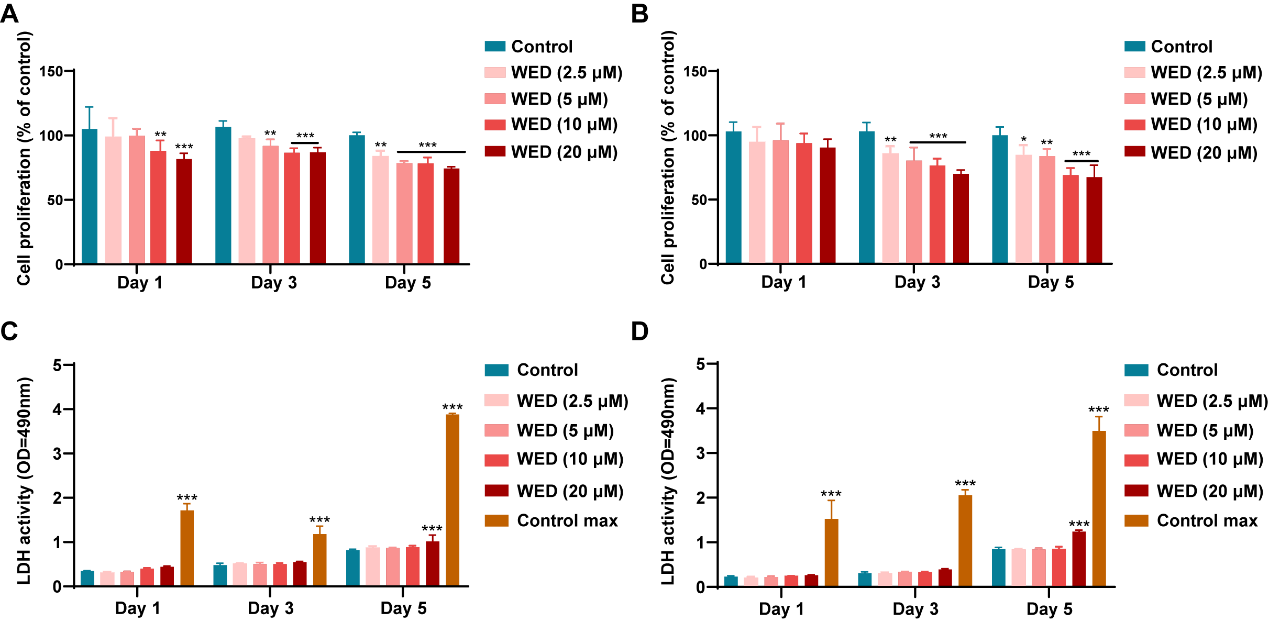


**Figure S1.** Cytotoxicity assessment of WED on HL60 and NB4 cells. A,B) CCK8 assay reveals the effects of WED (2.5, 5, 10, and 20 μM) on the proliferation of two cells (*n* = 5). C,D) Cytotoxicity of WED (2.5, 5, 10, and 20 μM) on the two cells, measured by LDH assay (*n* = 5). Data are presented as mean ± SD from at least three independent experiments. Statistical significance was determined using one-way ANOVA followed by Tukey’s post hoc test. **p* < 0.05, ***p* < 0.01, ****p* < 0.001, vs control.


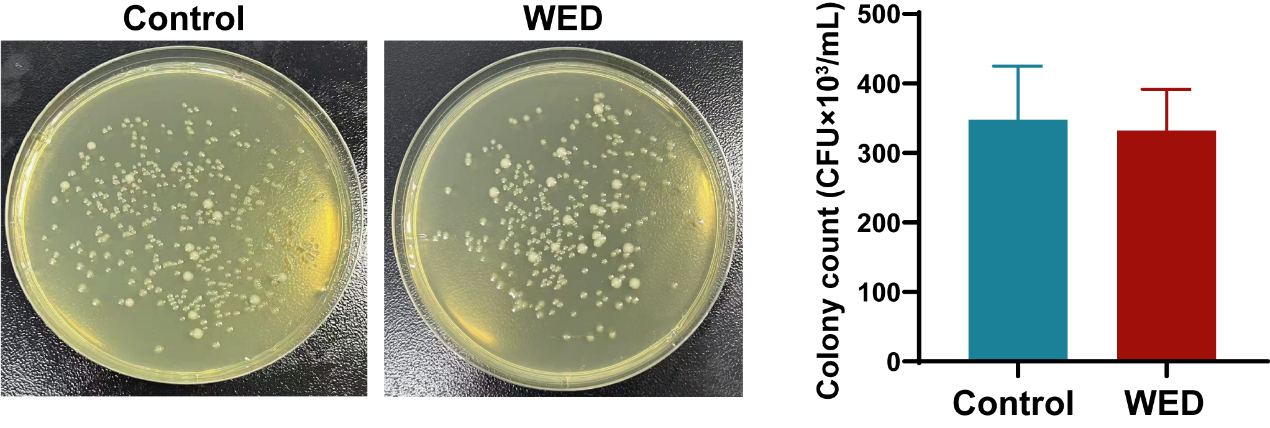


**Figure S2.** The bactericidal activity of WED itself (*n* = 3). Data are presented as mean ± SD from at least three independent experiments. Statistical significance was determined using an unpaired two-tailed Student’s *t*-test.


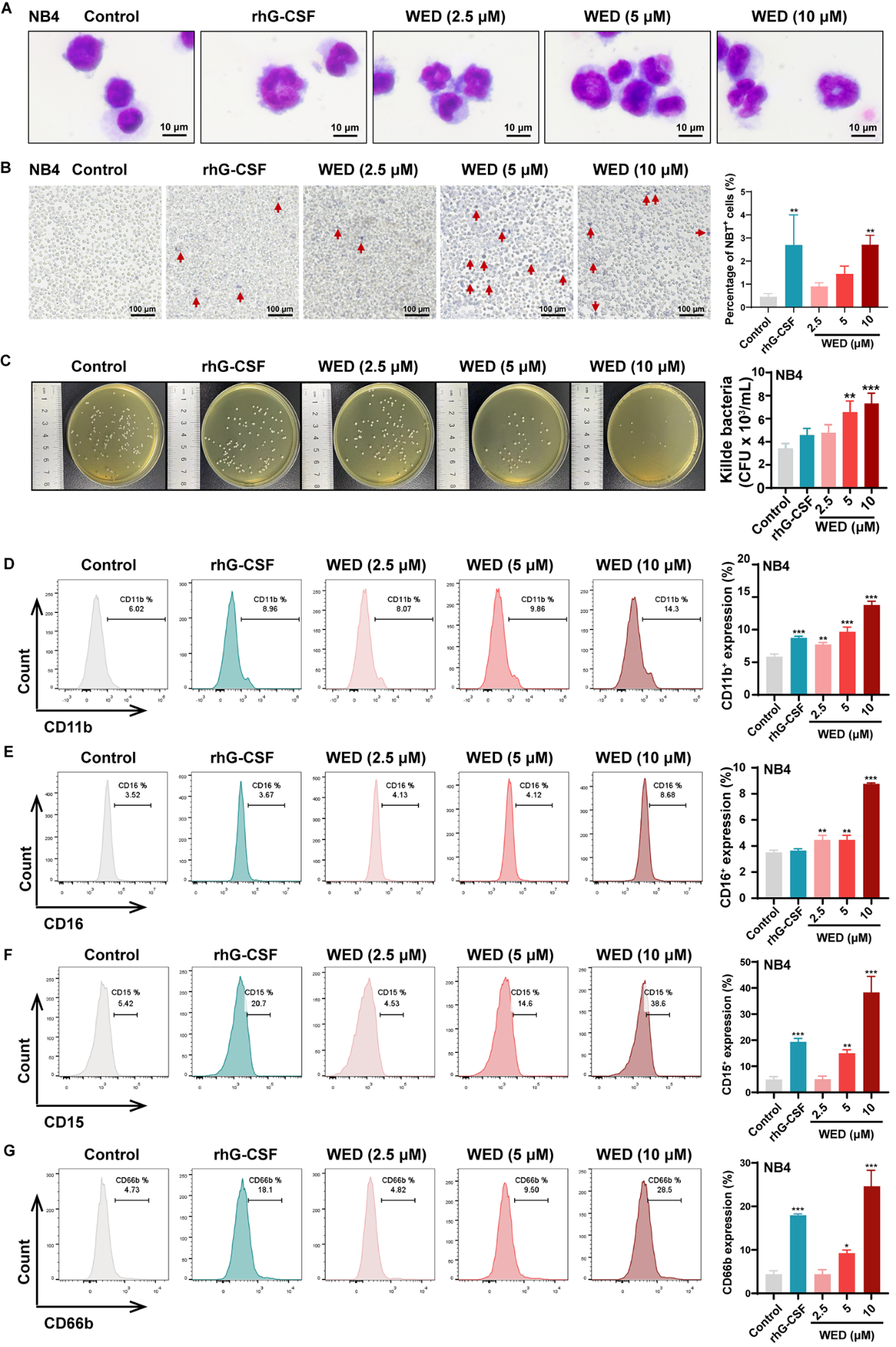


**Figure S3.** Effects of WED on neutrophil differentiation and bactericidal activity of NB4 cells. A) The cells are treated with the WED (2.5, 5 and 10 μM) for 5 days. Cell morphology is assessed using Giemsa staining. B) The cells are treated with the WED (2.5, 5 and 10 μM) for 5 days and incubated with NBT (1 mg/mL). Red arrows indicated NBT⁺ cells. Quantification of NBT⁺ cells. The percentage of NBT⁺ cells was calculated as the number of NBT⁺ cells divided by the total cell count per field (*n* = 3). C) Evaluation of bactericidal activity of WED (2.5, 5 and 10 μM) on *Staphylococcus aureus* (*S. aureus*). Histogram represents the killed bacteria of WED (2.5, 5 and 10 μM) on *S. aureus* (*n* = 3). D) Effects of WED (2.5, 5, and 10 μM) on CD11b expression (*n* = 3). E) Effects of WED (2.5, 5, and 10 μM) on CD16 expression (*n* = 3). F) Effects of WED (2.5, 5, and 10 μM) on CD15 expression (*n* = 3). G) Effects of WED (2.5, 5, and 10 μM) on CD66b expression (*n* = 3). Data are presented as mean ± SD from at least three independent experiments. Statistical significance was determined using one-way ANOVA followed by Tukey’s post hoc test. **p* < 0.05, ***p* < 0.01, ****p* < 0.001, vs control.


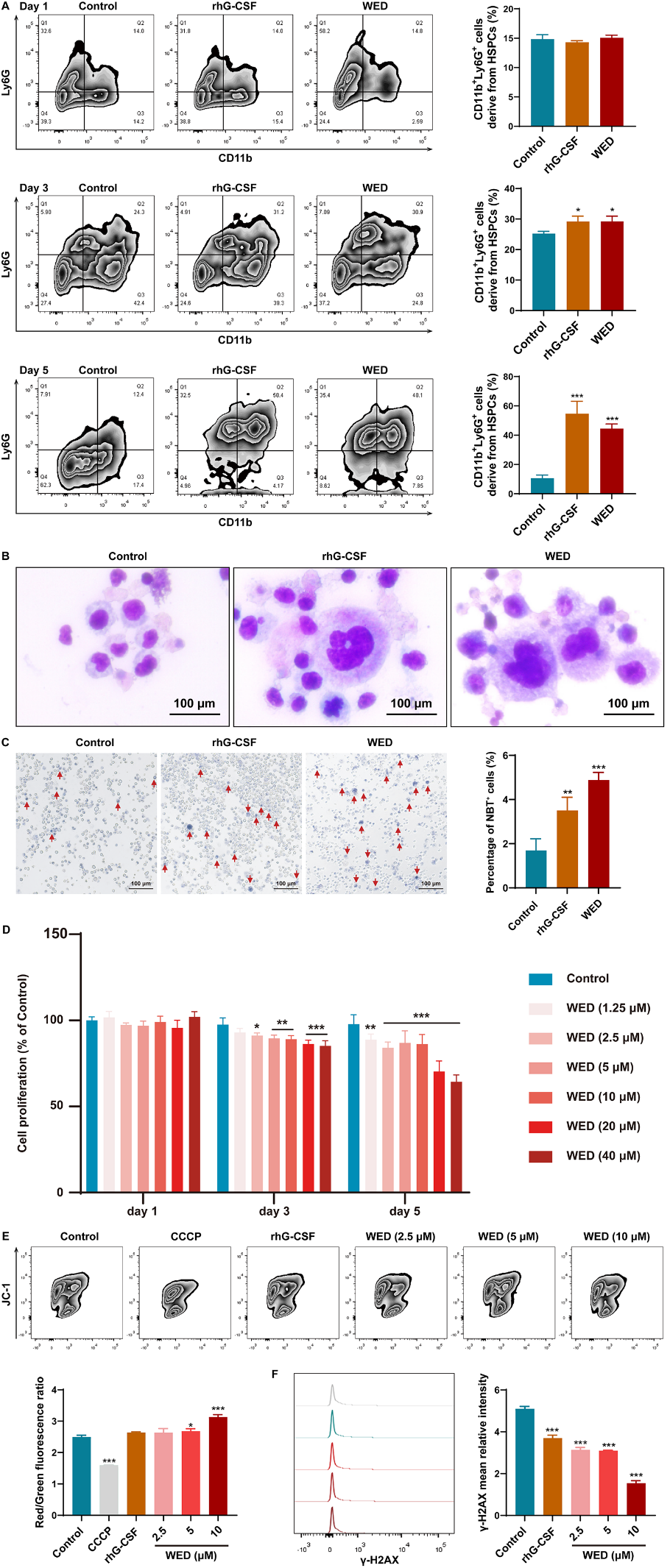


**Figure S4.** Effects of WED on neutrophil differentiation and function of normal mouse-derived HSPCs. A) Effects of WED (10 μM) or rhG-CSF (50 ng/mL) on CD11b and Ly6G expression. Representative flow cytometric plots and quantification of CD11b⁺Ly6G⁺ cells derived from mouse HSPCs on days 1, 3, and 5 (*n* = 3). B) Cell morphology observed by Giemsa staining. Representative images showing differentiated neutrophil morphology after 5 days of treatment with rhG-CSF (50 ng/mL) or WED (10 μM). C) Functional evaluation by NBT reduction assay. Red arrows indicate NBT⁺ cells. Quantification of NBT⁺ cells. The percentage of NBT⁺ cells was calculated as the number of NBT⁺ cells divided by the total cell count per field (*n* = 3). D) Effects of WED on proliferation of normal mouse-derived HSPCs (*n* = 3). E) JC-1 staining showing the effects of WED, rhG-CSF, and CCCP (positive control) on mitochondrial membrane potential. Quantification of the red/green fluorescence ratio is shown (*n* = 3). F) Flow cytometric analysis of γ-H2AX expression in HSPCs treated with rhG-CSF or WED. Histogram overlays and quantification of mean fluorescence intensity are presented (*n* = 3). Data are presented as mean ± SD from at least three independent experiments. Statistical significance was determined using one-way ANOVA followed by Tukey’s post hoc test. **p* < 0.05, ***p* < 0.01, ****p* < 0.001, vs control.


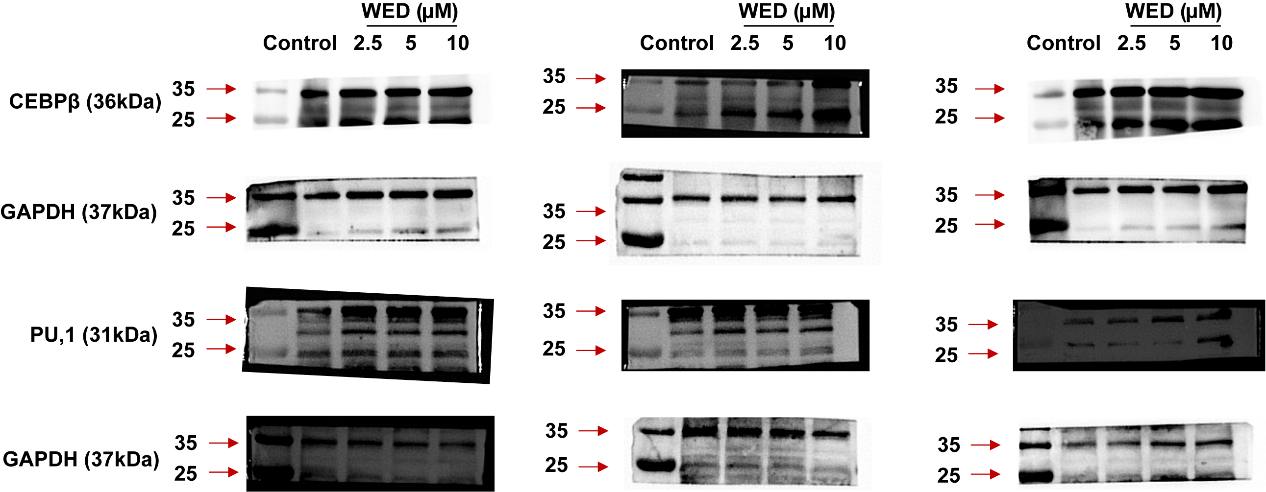


**Figure S5.** The source data for the western blot analysis presented in Figure 1.


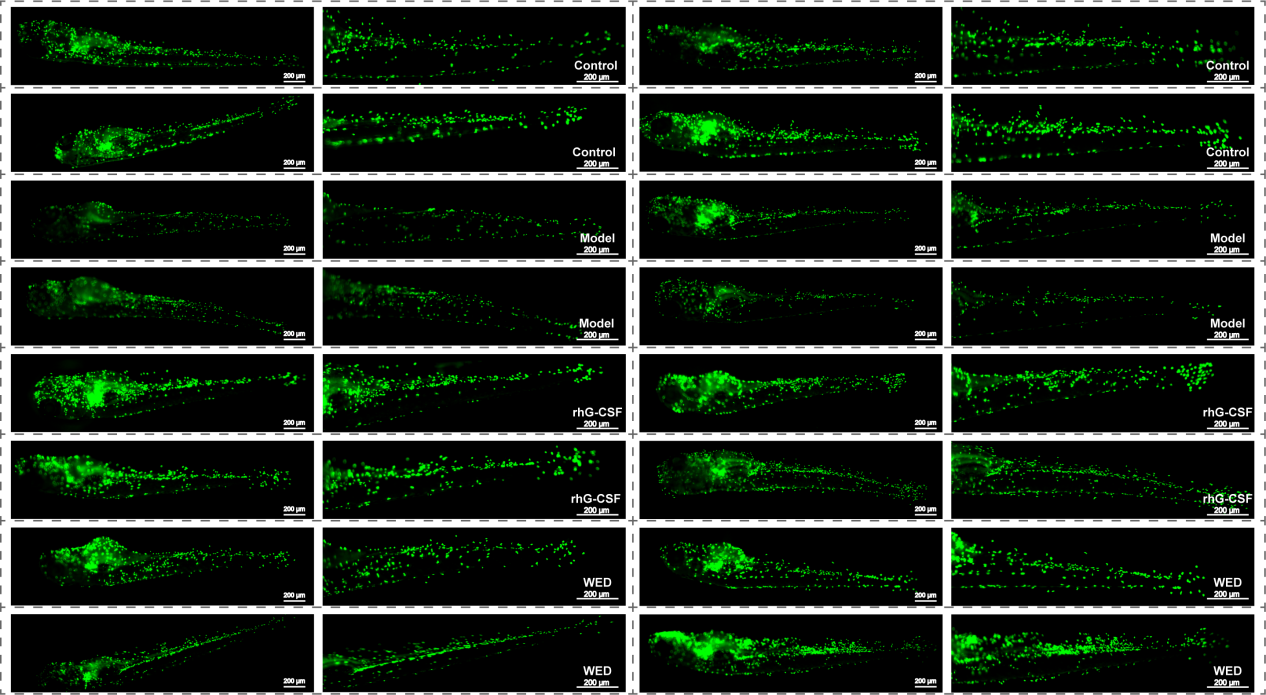


**Figure S6.** Radiation-induced neutropenic zebrafish is treated with WED (10 μM) or rhG-CSF (50 ng/mL). The zebrafish used is transgenic Tg (mpx: eGFP) zebrafish, expressing green fluorescent protein specifically in neutrophils.


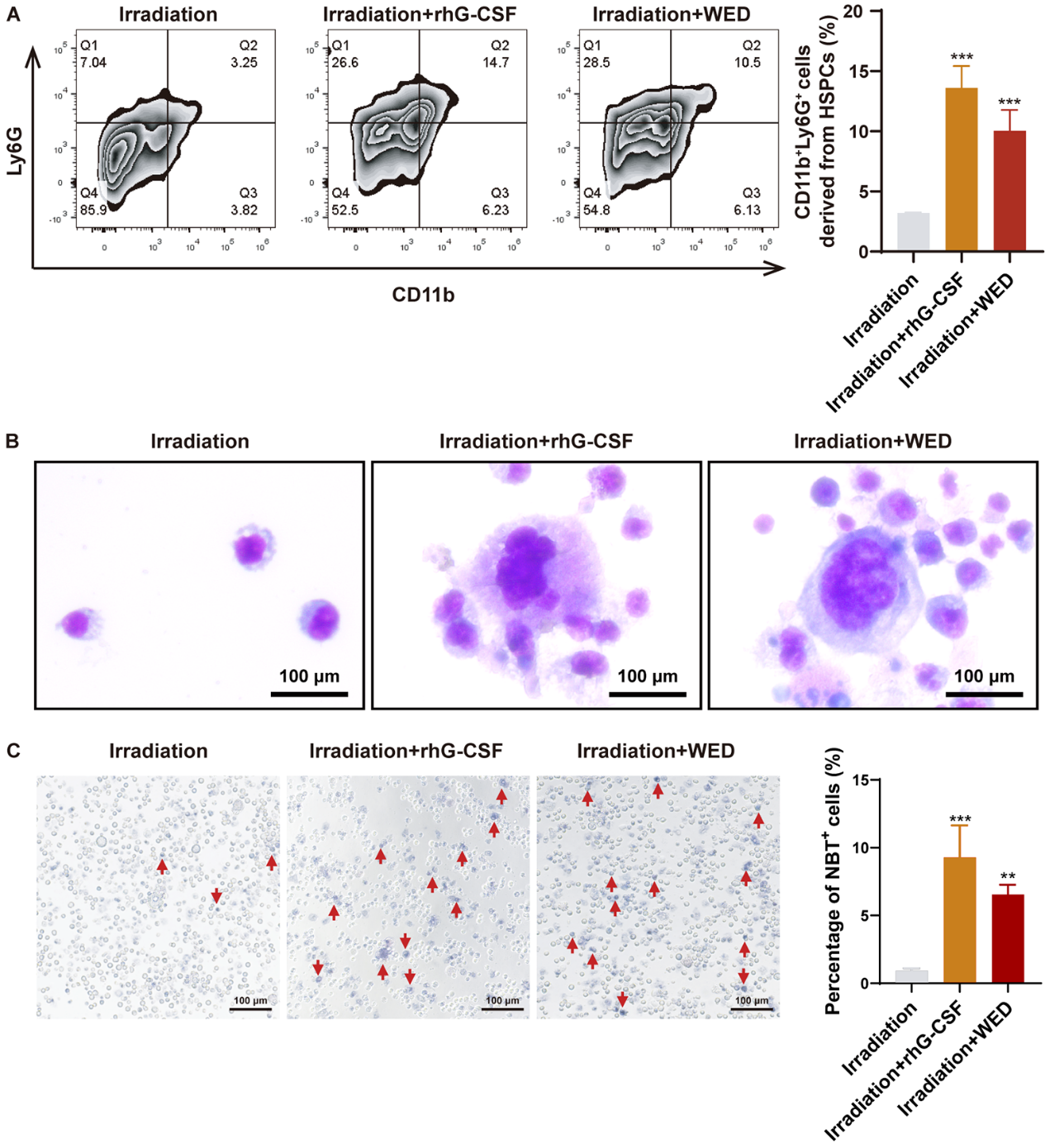


**Figure S7.** Effects of WED on neutrophil differentiation and function of irradiation mouse-derived HSPCs. A) Effects of WED (10 μM) or rhG-CSF (50 ng/mL) on CD11b and Ly6G expression (*n* = 3). B) Cell morphology observed by Giemsa staining. C) Functional evaluation by NBT reduction assay. Red arrows indicate NBT⁺ cells. Quantification of NBT⁺ cells. The percentage of NBT⁺ cells was calculated as the number of NBT⁺ cells divided by the total cell count per field (*n* = 3). Data are presented as mean ± SD from at least three independent experiments. Statistical significance was determined using one-way ANOVA followed by Tukey’s post hoc test. ***p* < 0.01, ****p* < 0.001, vs irradiation group.


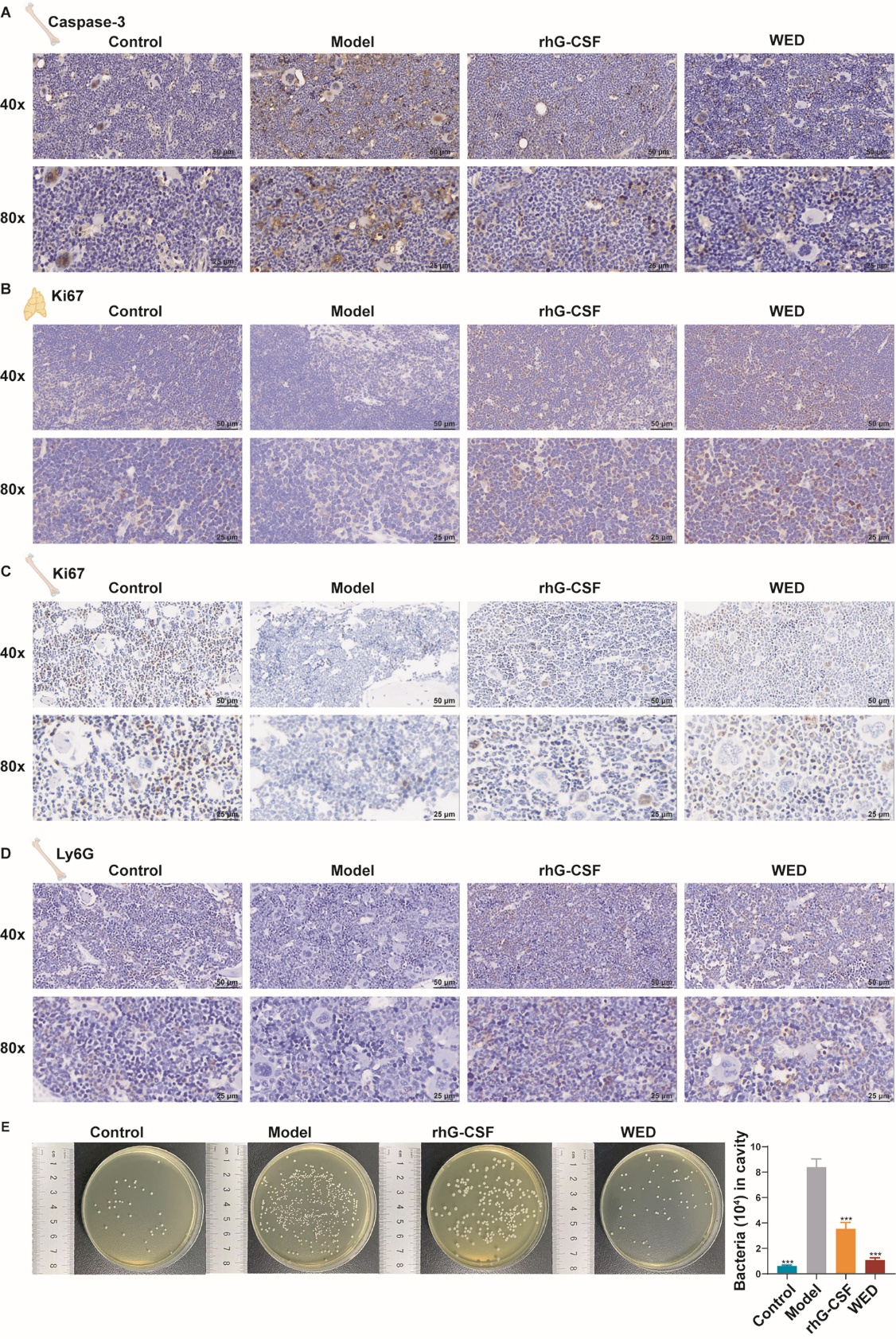


**Figure S8.** Effects of WED on Caspase-3, Ki67 and Ly6G expression, and neutrophil bactericidal activity in neutropenic mice. A) IHC analysis of Caspase-3 expression in bone marrow in each group. Brown staining indicates Caspase-3-positive cells. B,C) IHC analysis of Ki67 expression in thymocytes and bone marrow in each group. Brown staining indicates Ki67-positive cells. D) IHC analysis of Ly6G expression in bone marrow. Brown staining represents Ly6G-positive cells. E) The bacterial killing ability of WED on *S. aureus*. Sixteen hours post-infection, the viable bacterial colonies within the cavity are counted (*n* = 3). Data are presented as mean ± SD from at least three independent experiments. Statistical significance was determined using one-way ANOVA followed by Tukey’s post hoc test. ****p* < 0.001, vs model.


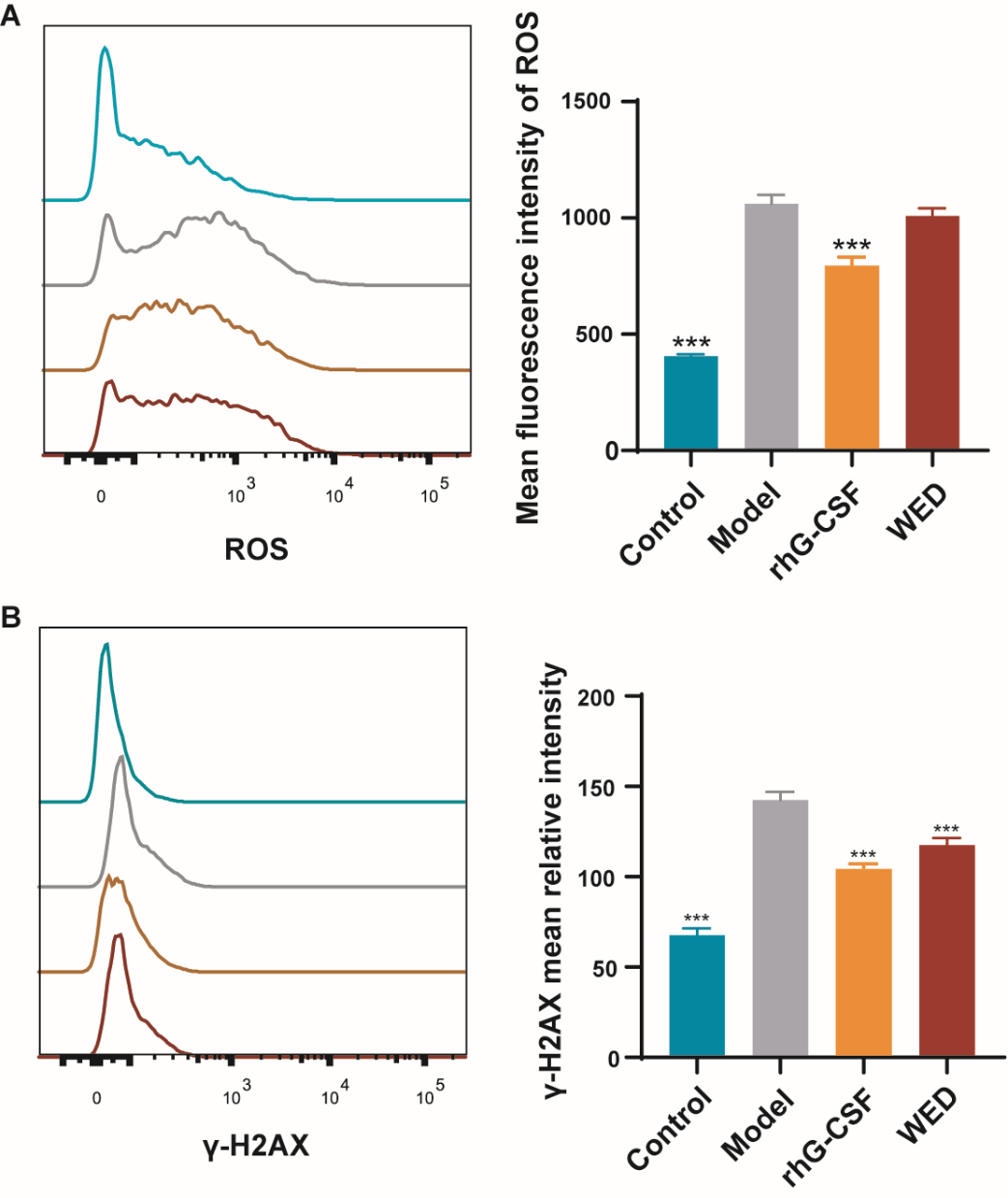


**Figure S9.** Effect of WED on irradiation-induced injury. A) Analysis of ROS levels by flow cytometry in each group (*n* = 3). B) Analysis of γ-H2AX expression by flow cytometry in each group (*n* = 3). Data are presented as mean ± SD from at least three independent experiments. Statistical significance was determined using one-way ANOVA followed by Tukey’s post hoc test. ****p* < 0.001, vs model.


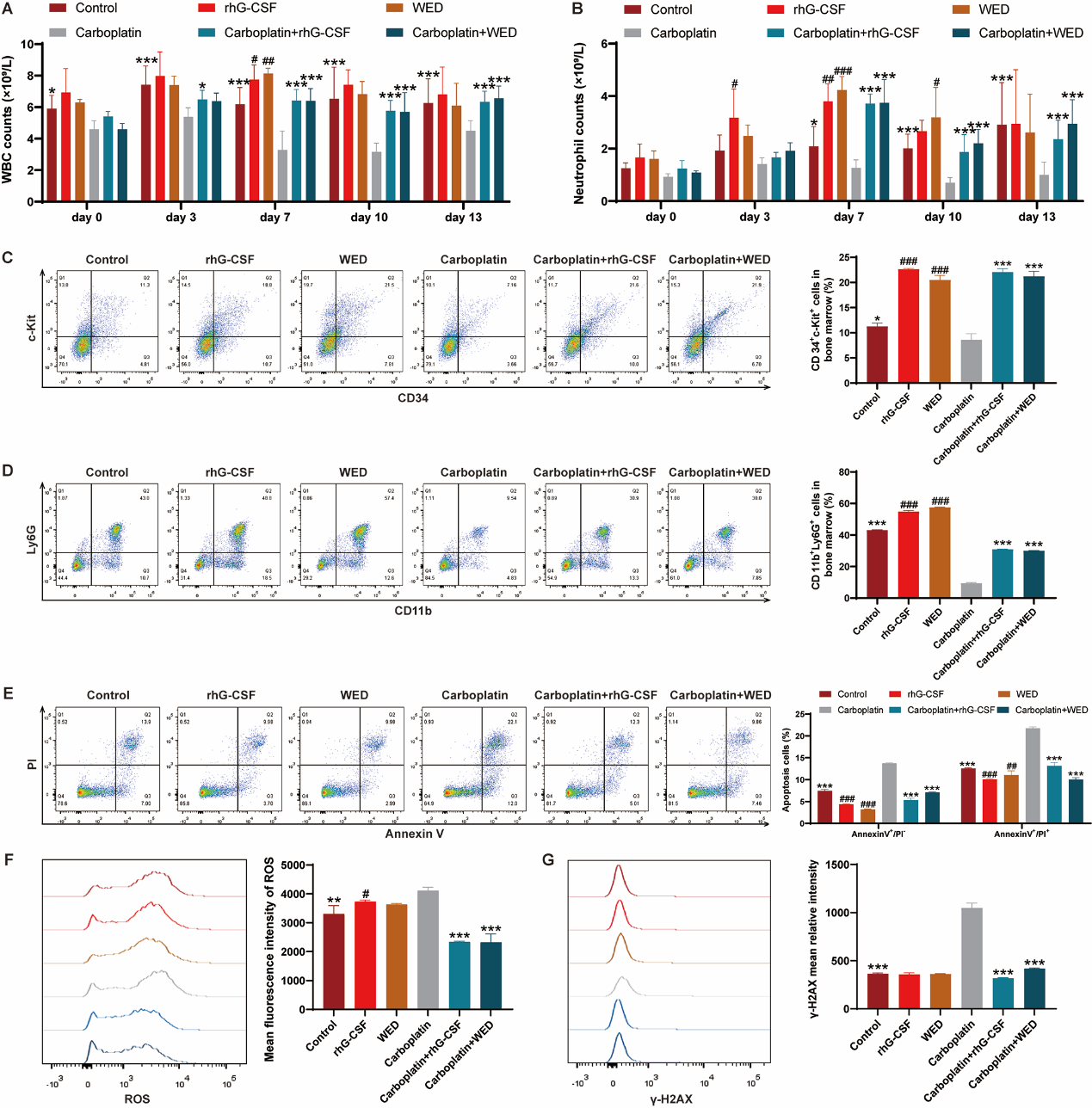


**Figure S10.** Therapeutic effects of WED on carboplatin-induced neutropenia in mice. A) Effects of WED (5 mg/kg) or rhG-CSF (25 μg/kg) on WBC counts in carboplatin-induced neutropenic mice on days 0, 3, 7, 10, and 13 after treatment (*n* = 8). B) Effects of WED WED (5 mg/kg) or rhG-CSF (25 μg/kg) on neutrophil counts in peripheral blood at the indicated time points (*n* = 8). C) Flow cytometric analysis of CD34 and c-Kit expression in bone marrow cells from each group. The histogram shows the proportion of CD34⁺c-Kit⁺ cells in bone marrow (*n* = 3). D) Flow cytometric analysis of CD11b and Ly6G expression in bone marrow cells from each group. The histogram shows the proportion of CD11b⁺Ly6G⁺ cells in bone marrow (*n* = 3). E) Flow cytometric analysis of bone marrow cell apoptosis using Annexin V/PI staining. The histogram shows the proportions of early apoptotic (Annexin V⁺/PI⁻) and late apoptotic (Annexin V⁺/PI⁺) cells (*n* = 3). F) Measurement of intracellular ROS levels in bone marrow cells by flow cytometry. The histogram shows the mean fluorescence intensity of ROS (*n* = 3). G) Flow cytometric analysis of γ-H2AX expression in bone marrow cells. Histogram overlays and quantification of mean relative fluorescence intensity are shown (*n* = 3). Data are presented as mean ± SD from at least three independent experiments. Statistical significance was determined using one-way ANOVA followed by Tukey’s post hoc test. **p* < 0.05, ***p* < 0.01, ***p* < 0.001, vs carboplatin group; ^#^*p* < 0.05, ^##^*p* < 0.01, ^###^*p* < 0.001, vs control group.


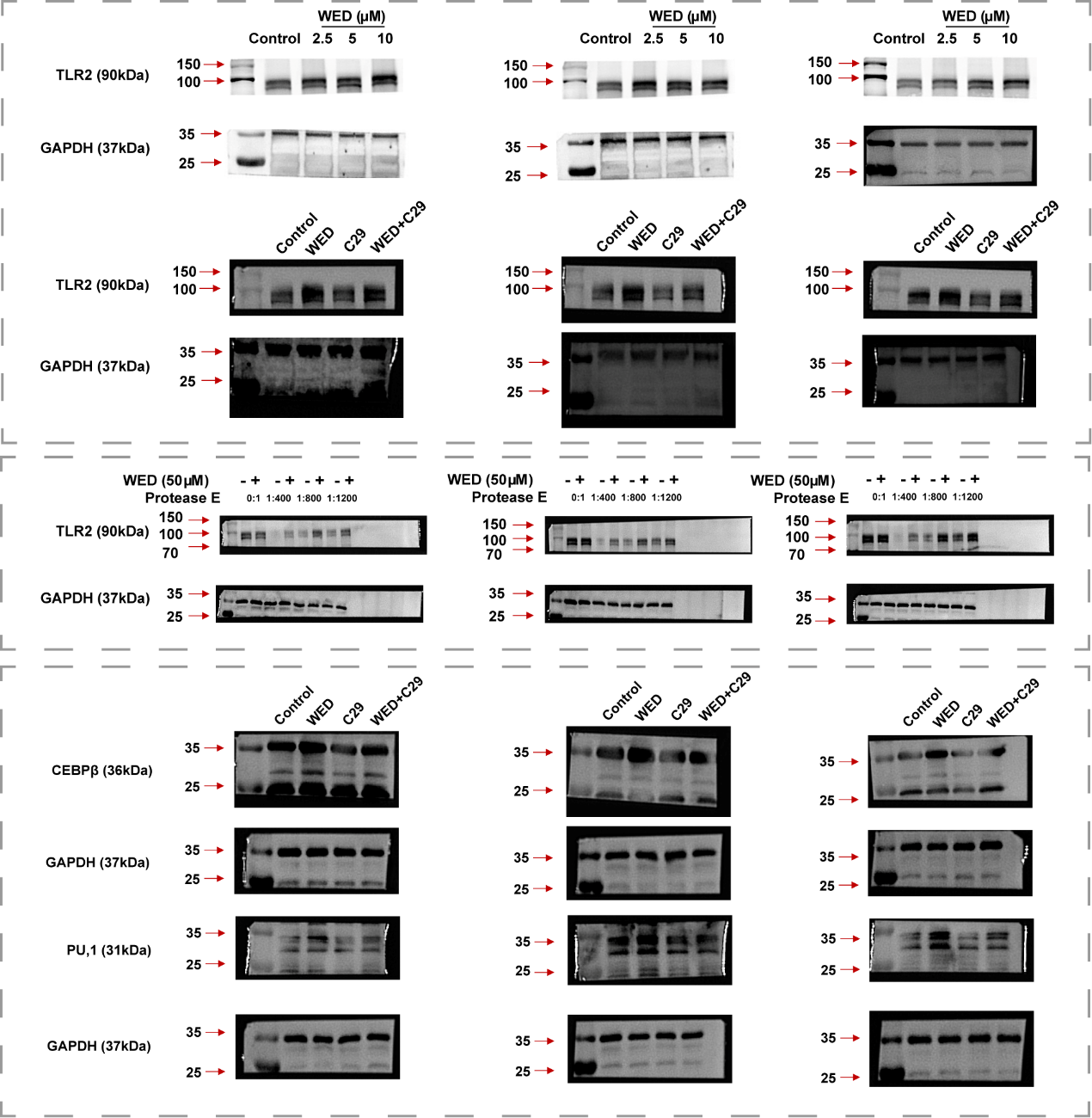


**Figure S11.** The source data for the western blot analysis presented in Figure 7.


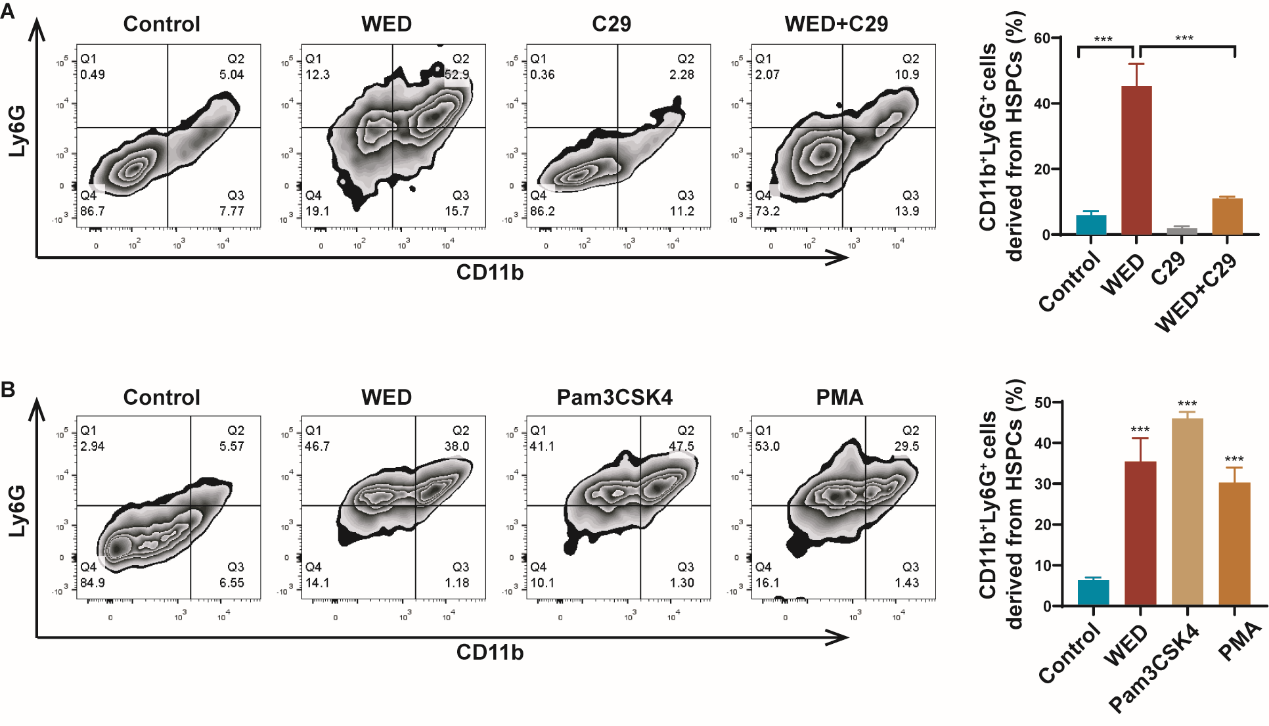


**Figure S12.** Essential roles of the TLR2 and MAPK signaling pathways in neutrophil differentiation of mouse-derived HSPCs. A) Effect of the TLR2 inhibitor C29 (50 μM) on neutrophil differentiation (*n* = 3). B) Effects of the TLR2 agonist Pam3CSK4 (100 ng/mL) and the MAPK pathway activator PMA (10 ng/mL) on neutrophil differentiation (*n* = 3). Data are presented as mean ± SD from at least three independent experiments. Statistical significance was determined using one-way ANOVA followed by Tukey’s post hoc test. ****p* < 0.001, vs the corresponding control groups.


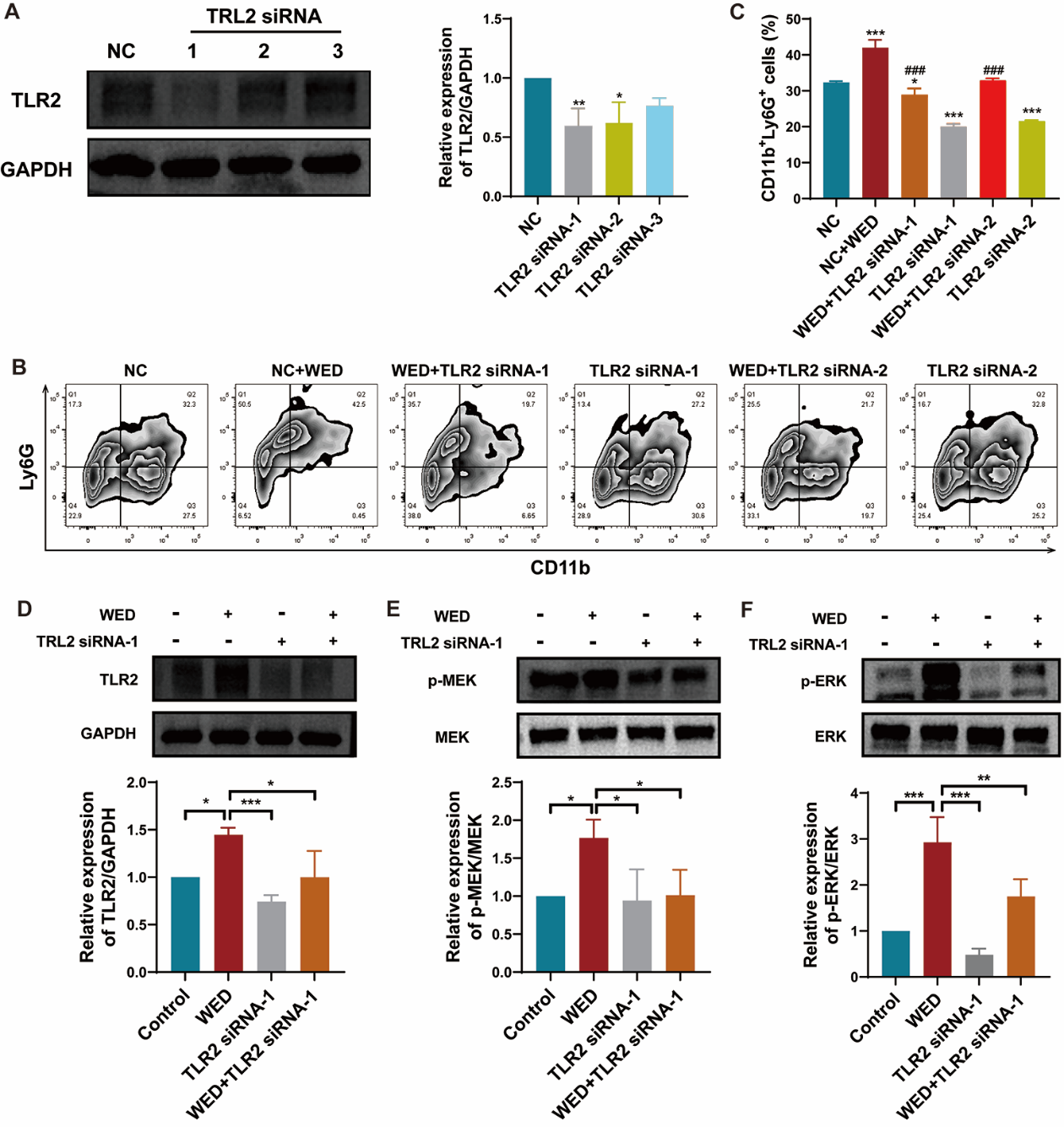


**Figure S13.** Effects of TLR2 knockdown on WED-induced neutrophil differentiation and MAPK pathway activation in mouse HSPCs. A) Western blot validation of three TLR2 siRNAs in primary mouse HSPCs. TLR2 expression was normalized to GAPDH, and siRNA-1 and siRNA-2 showed the most efficient knockdown (*n* = 3). B) Flow cytometric analysis of CD11b and Ly6G expression in HSPCs transfected with TLR2 siRNA-1 or siRNA-2 and treated with WED (10 µM) for 5 days. Representative plots and quantification of CD11b⁺Ly6G⁺ cells are shown (*n* = 3). C) Quantification of CD11b⁺Ly6G⁺ cell percentages from each group (*n* = 3). D) Western blot analysis of TLR2 expression in HSPCs after TLR2 siRNA transfection and WED treatment (*n* = 3). E,F) Western blot analysis of MEK/ERK pathway activation. Representative blots and quantification of p-MEK/MEK and p-ERK/ERK levels in each group (*n* = 3). Data are presented as mean ± SD from at least three independent experiments. Statistical significance was determined using one-way ANOVA followed by Tukey’s post hoc test. **p* < 0.05, ***p* < 0.01, ***p* < 0.001, vs corresponding control groups; ^###^*p* < 0.001, vs NC+WED group.


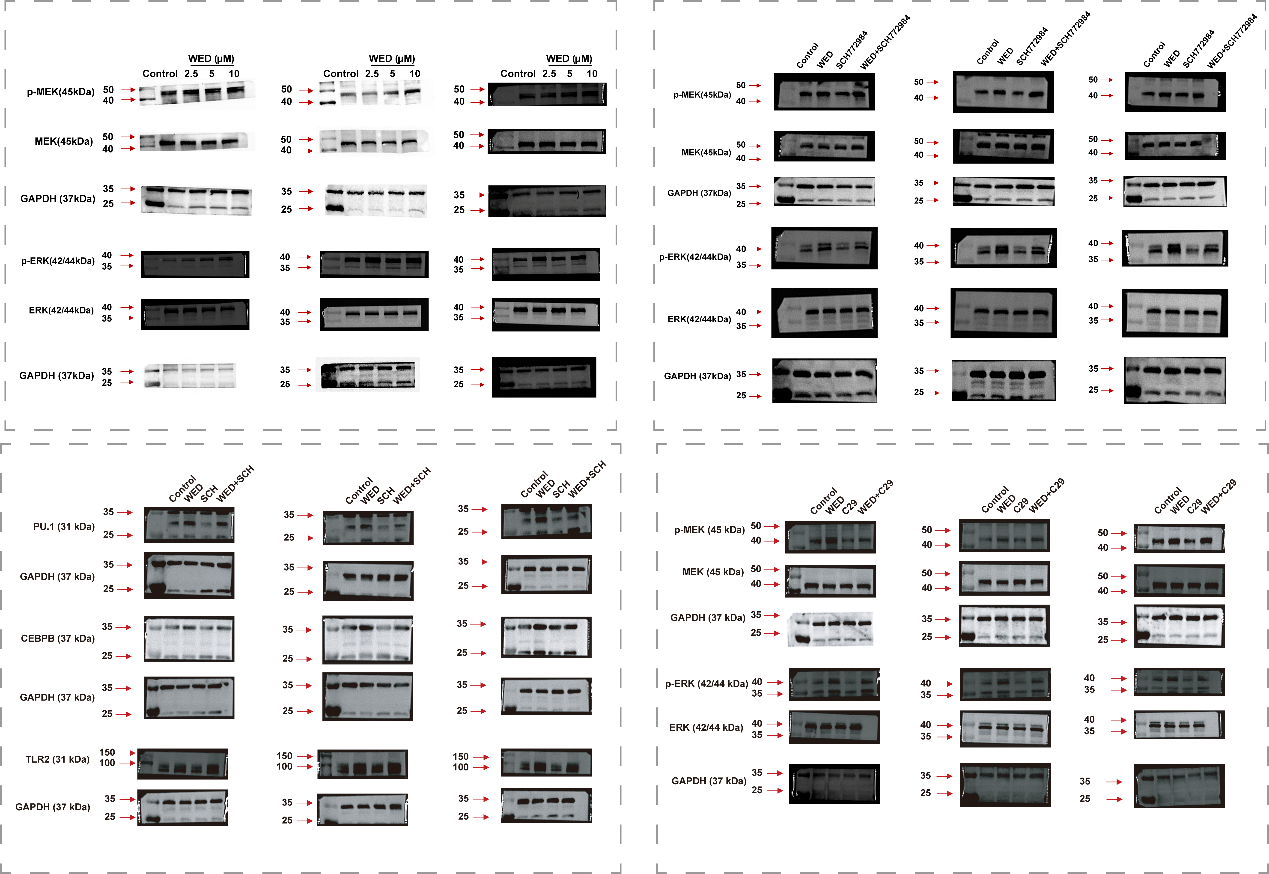


**Figure S14.** The source data for the western blot analysis presented in Figure 8.


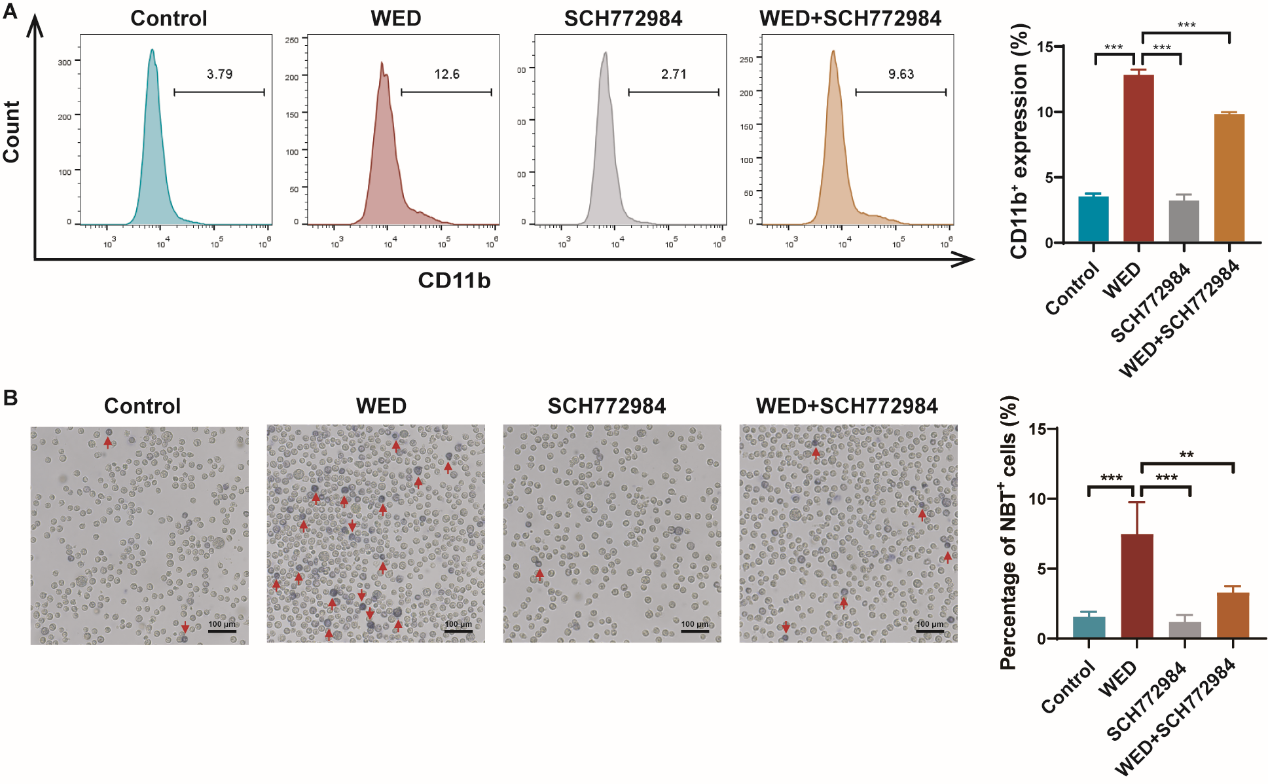
 **Figure S15.** WED promotes neutrophil differentiation through activation of the MEK/ERK pathway. A) Flow cytometry analysis of CD11b expression in HL60 cells treated with WED (10 μM) and SCH772984 (2.5 μM) for 5 days (*n* = 3). B) Detection of NBT reducing activity after treatment with WED (10 μM) and SCH772984 (2.5 μM) for 5 days in HL60 cells. Red arrows indicate NBT⁺ cells. Quantification of NBT⁺ cells. The percentage of NBT⁺ cells was calculated as the number of NBT⁺ cells divided by the total cell count per field (*n* = 3). Data are presented as mean ± SD from at least three independent experiments. Statistical significance was determined using one-way ANOVA followed by Tukey’s post hoc test. ****p* < 0.001, vs WED group.


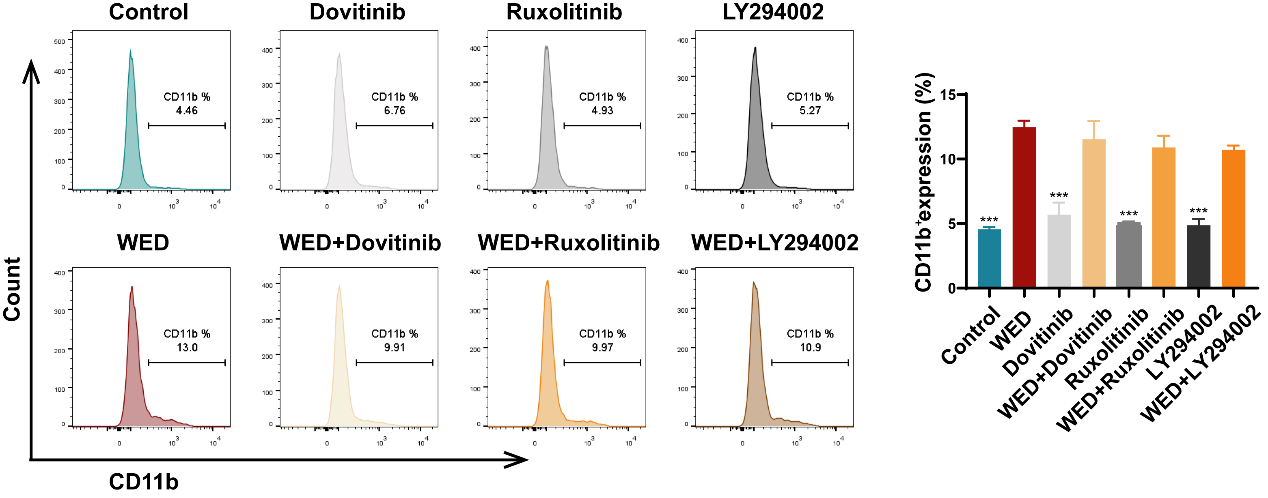


**Figure S16.** The effects of LY294002, Ruxolitinib, and Dovitinib on WED-induced neutrophil differentiation in HL60 cells. Flow cytometry analysis of CD11b expression in cells treated with WED (10 μM), Dovitinib (0.8 μM), LY294002 (8 μM), and Ruxolitinib (30 μM), for 5 days (*n* = 3). Data are presented as mean ± SD from at least three independent experiments. Statistical significance was determined using one-way ANOVA followed by Tukey’s post hoc test. ****p* < 0.001, vs WED group.


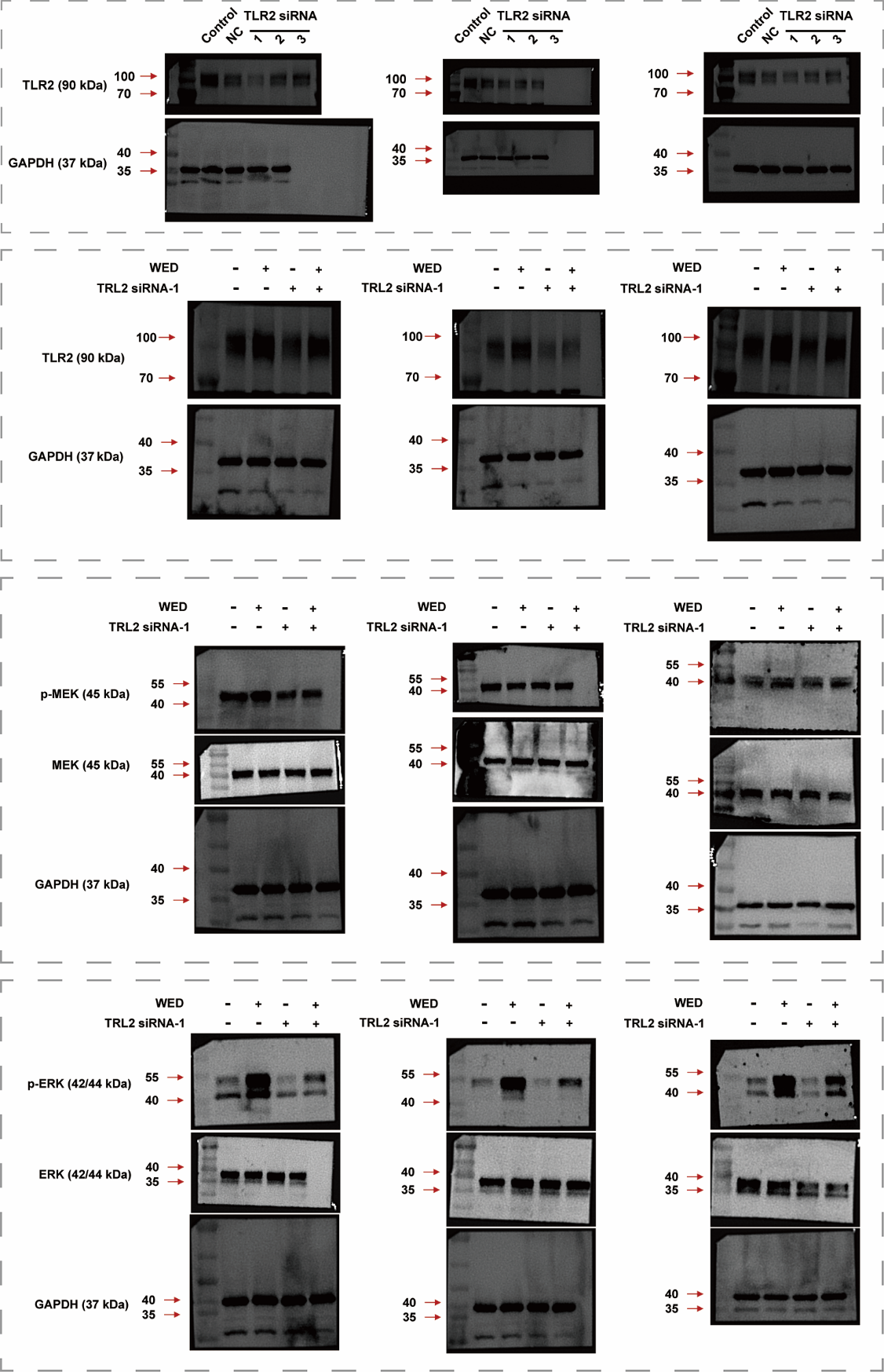


**Figure S17.** The source data for the western blot analysis presented in Figure S13.


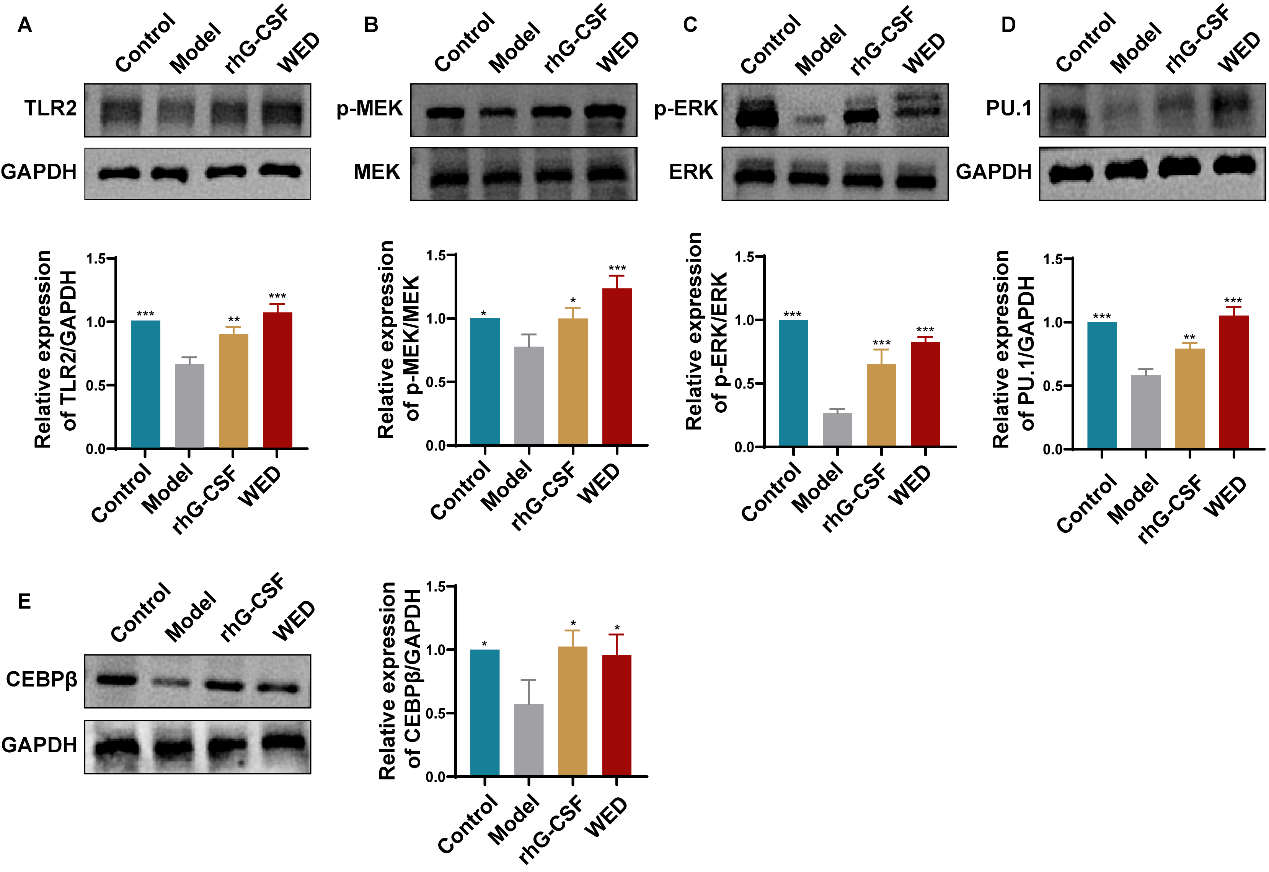


**Figure S18.** Western blot analysis of TLR2, MEK, ERK, PU.1 and CEBPβ expression in bone marrow cells of neutropenic mice. A-E) Western blot analysis of the expression of TLR2, p-MEK, p-ERK, PU.1, CEBPβ after WED (5 mg/kg) or rhG-CSF (25 μg/kg) treatment. The histograms represent the expression of proteins of each group (*n* = 3). Data are presented as mean ± SD from at least three independent experiments. Statistical significance was determined using one-way ANOVA followed by Tukey’s post hoc test. **p* < 0.05, ***p* < 0.01, ****p* < 0.001, vs model.


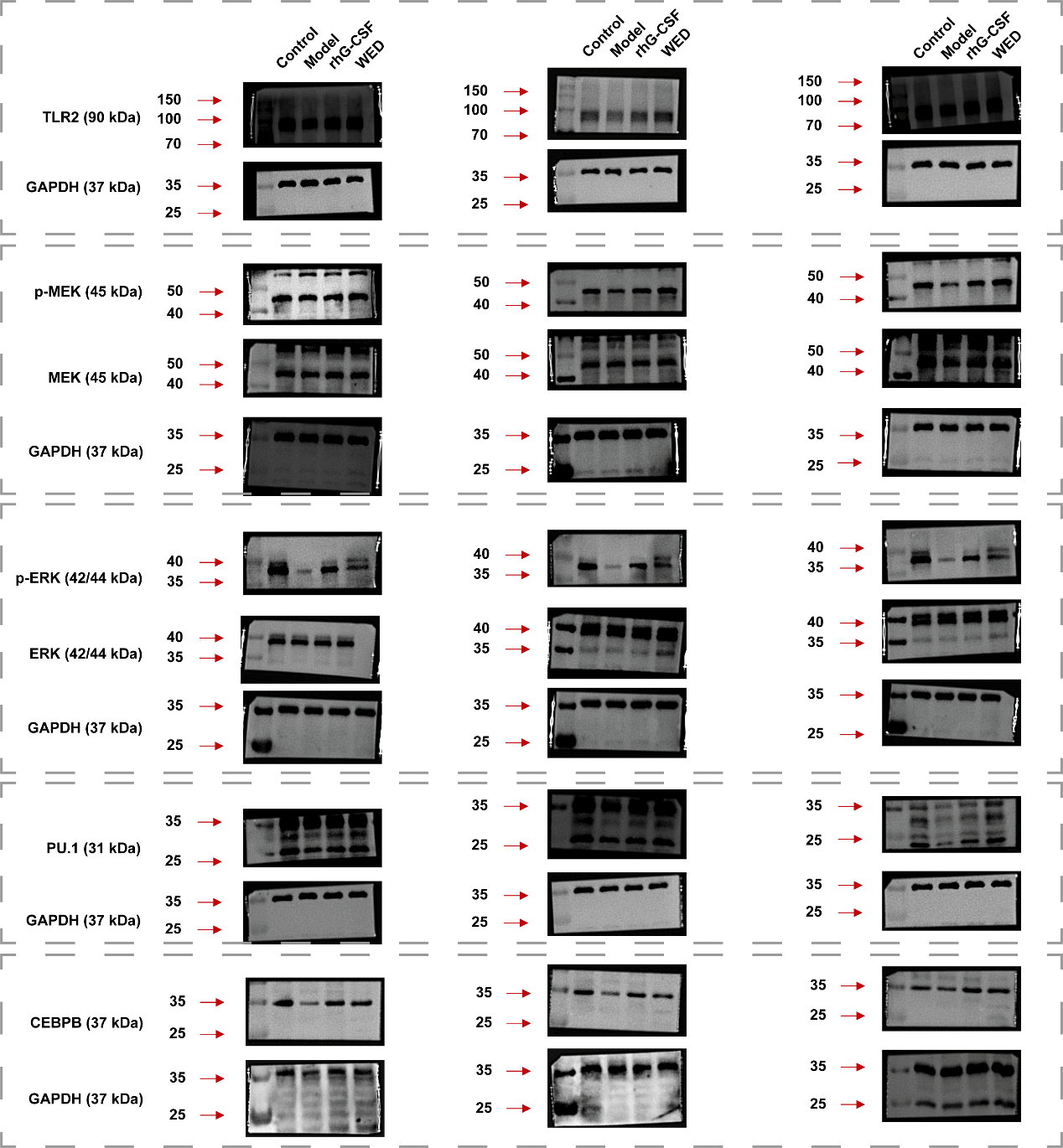


**Figure S19.** The source data for the western blot analysis presented in Figure S18.
